# New Approaches to Assess Food Web Stability in Aquatic Ecosystems: A Case Study on Baiyangdian Lake

**DOI:** 10.1002/ece3.71934

**Published:** 2025-08-08

**Authors:** Yong Zeng, Wei Yang, Yanwei Zhao

**Affiliations:** ^1^ State Key Laboratory of Heavy Oil Processing, Beijing Key Laboratory of Oil & Gas Pollution Control, College of Chemical Engineering and Environment China University of Petroleum Beijing China; ^2^ State Key Laboratory of Water Environment Simulation, School of Environment Beijing Normal University Beijing China

**Keywords:** Baiyangdian Lake, detritus food web, interaction strength, stability analysis

## Abstract

Interactions between species and detritus in aquatic ecosystems involve unassimilated food, non‐predator mortality, and complex trophic relationships, making it challenging to quantify interaction strengths. This study utilized classic and revised Lotka–Volterra equations, combined with the food web of Baiyangdian Lake, to develop methods for measuring interaction strengths in a phytoplankton‐based and a detritus food web. The analysis relied on three types of species–detritus interactions and outputs from an Ecopath model (1958–2019). Loop weight and Diagonal strength (*S*) were employed to assess stability. Lighter loops weight and lower *S* value indicate higher stability. From 1958 to 2009, the stability of Baiyangdian Lake was limited by a three‐link omnivorous loop: Detritus > zooplankton > filter‐feeding fish. As the predator–prey biomass ratio (filter‐feeding fish/detritus) increased, instability increased, and vice versa. However, the new loop (detritus > zooplankton > phytoplankton) and corresponding new predator–prey biomass ratios (zooplankton/detritus) resulted in stability from 2009 to 2019. It inferred dominant top‐down trophic cascade effects changed to dominant bottom‐up trophic cascade effects. Besides focusing on the heaviest loop weight, it was necessary to examine the heavier loops that may have a chance of evolving into the heaviest ones following catastrophic or long‐term perturbations to the food web. To facilitate management, a geometric mean ratio of predator‐to‐ prey biomass ${\left(\frac{B_{C}}{B_{P}}\right)}_{t}$ was proposed as a simplified indicator. This metric correlates with diagonal strength (*R* ^2^ = 0.6645) and offers a practical tool for early‐warning assessments of food web stability, despite its moderate precision. This study highlights the importance of integrating detritus dynamics into stability analyses and using loop weight analysis to identify critical trophic interactions. The proposed empirical indicators provide a bridge between theoretical models and ecosystem management practices.

## Graphical abstract

A method for calculating the interaction strength of a phytoplankton‐based and a detritus food web. A geometric mean ratio of predator‐to‐prey biomass ${\left(\frac{B_{C}}{B_{P}}\right)}_{t}$ as an alternative indicator for food web stability. The changes in the stability of the BYD Lake from the 1950s to the 2010s indicate that two steady‐state transformations have occurred.

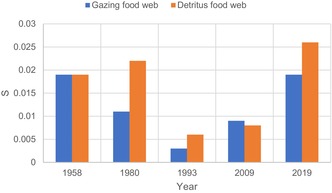

## 1 Introduction

Food web stability is defined as the ability of a food web system to maintain its previous state in the face of disturbance (Zhang, Yi, et al. 2023). The stability of food web dynamics refers to whether the population dynamics of a multi‐species system are asymptotically stable in a strict mathematical sense. When the biomass of food web species returns to the initial equilibrium state after being disturbed, the food web system is considered stable. The stability analysis of food web dynamics refers to judging whether the eigenvalue with the largest real part of the Jacobian (community) matrix of the linear multi‐species differential equation group is greater than zero. If the real part is positive, the equilibrium is unstable; if the real part is negative, the system is stable and will converge back to a stable state (May 1972; Allesina and Tang 2015; Landi et al. 2018). Food web stability plays a prominent role in ecological services and safety (Kang et al. 2022). Stability assessments of food webs can serve as an early indicator to identify and track perturbation‐induced changes in ecosystems and the thresholds beyond which they lose stability (Brito et al. 2024).

Since Robert May (1972) first proposed a method of analyzing the asymptotic local stability of large ecological systems, efforts to quantify the strength of species interactions have become an important focus in research on ecological communities (Novak 2010). Following de Ruiter et al. (1995), interaction strengths ($\alpha_{\mathrm{ij}}$) are defined as the entries of a Jacobian or community matrix, which is the direct effect of the average species *j* individual on species *i*'s population growth rate. The local stability of an equilibrium point can then be determined by inspecting the eigenvalues of this matrix. The equilibrium point is locally stable if the largest eigenvalue of the Jacobian community matrix has a negative real part (Emmerson and Yearsley 2004). As an abstract concept, empirical estimates of the interaction strength remain challenging because of the lack of congruence in definitions, the complexity of measuring methods and difficulties designing control experiments (Laska and Wootton 1998; Novak and Wootton 2010). Despite their importance, there are few methods of estimating interaction strength (Laska and Wootton 1998; Wootton and Emmerson 2005). One basic approach is to assume that the elements of interaction strength are randomly distributed variables (Allesina and Tang 2015; Stone 2018) with hypothetical values ranging from −1 to 1. Monte Carlo simulations are then used for a theoretical analysis (Mougi and Kondoh 2012; Stouffer and Bascompte 2011; Rozdilsky and Stone 2010). Another approach involves estimating the interaction strength according to the energy flow between species in a food web based on models of mass or energy balance (Hunt et al. 1987; de Ruiter et al. 1995; McCann et al. 1998; Teng and Mccann 2004; Rooney et al. 2006; Kuiper et al. 2015; Jacquet et al. 2016; Landi et al. 2018; Hu et al. 2022). The intraspecific interaction strength ($\alpha_{\mathrm{ii}}$) is difficult to calculate directly, due to a lack of observed and experimental data. However, it can be expressed as the proportion of specific mortality caused by intraspecific competition. This value represents the minimum intraspecific interaction needed for matrix stability. In order to focus on energy flow in critical links, loop weight analysis was proposed by Neutel et al. (2002), in which the maximum of all the loop weights in a community matrix is used as an approximation of the absolute value of intraspecific interaction sufficient for matrix stability. A loop is a closed chain of trophic links, and loop weight is defined as the geometric mean of the absolute values of the interaction strengths in the loop. Zhang, Yi, et al. (2023) simulated the current dynamics of food web stability under different phosphorus loads for Lake Baiyangdian in China using loop weight analysis. They found that the loop of detritus, diatoms, and zooplankton affected food web stability.

Although great progress has been made in the quantification of interaction strength matrices, to calculate real food webs, many types and large quantities of data are required, for example, data on the biomass, diet composition, predation, non‐predation, intrinsic mortality rates, assimilation efficiency, and production efficiency of each species. Certain data are difficult to obtain from observations and experiments. This shortcoming affects calculations of interaction strengths and stability analysis. Therefore, more convenient and practical calculation methods and easier access to data are urgently needed. The Ecopath modeling framework is one such approach. It provides alternative parameters to facilitate calculations of the interaction strength in food webs (DeAngelis et al. 1989; Bascompte et al. 2005; Jacquet et al. 2016).

Traditional approaches to studying food webs emphasize the transfer of local primary productivity in the form of living plant matter across trophic levels (Pękalski and Szwabiński 2014). Consequently, existing theories on food webs have largely neglected detritus‐based systems and instead focused on phytoplankton‐based food webs with living plant biomass as the energy source for first‐level consumers (Moore et al. 2004). The detritus food web operates differently, with detritus serving as both an energy source for consumers of many trophic levels and a nutrient reservoir for plants. The amount of energy flowing through the detrital pathway can equal or exceed that of the phytoplankton grazing pathway. As both an energy source and nutrient reservoir, detritus plays an important role in nutrient cycling and food web dynamics (DeAngelis et al. 1989; Moore et al. 1993; Pękalski and Szwabiński 2014). In many ecosystems, detrital chains are the major pathway of energy flow and have a critical stabilizing effect on trophic dynamics (Kuiper et al. 2015; Neutel et al. 2007). The inclusion of detrital heterogeneity in models of food web dynamics has attracted the attention of researchers (Moore et al. 2004). Literature shows that the predictive power of theories on food webs may be questioned if they do not include the detritus path. Thus, food web theories should include the detritus functional group and its relevant energy paths before applying them to real ecosystems and drawing conclusions (Moore et al. 2004). The interaction strength between a functional group and detritus can be derived from revised Lotka–Volterra equations for detritus, very similar to those of predator–prey interactions (Moore et al. 1993; de Ruiter et al. 1995). However, previous studies are mostly theoretical, and the move from theory to practice remains ambiguous, perhaps due to its complexity, in particular with regard to calculating the interaction strength between functional group species and detritus. Therefore, it is necessary to comprehensively reexamine all bioenergetic processes between functional groups and between functional group species and detritus to bridge the gap between theory and practice.

To fill this gap, we proposed an approach to measuring the interaction strength of the detritus food web. The interaction strength between functional group species and detritus was identified starting from a theoretical analysis of the generalized form of the Lotka–Volterra equations, as well as a revised form that included detritus recycling. Then, we analyzed the stability of Baiyangdian (BYD) Lake in northern China from the 1950s to the 2020s, based on the proposed measuring approaches with the help of the energetic output of the BYD Lake Ecopath model (Zeng et al. 2021) over five periods. The diagonal strength and loop weight analysis (de Ruiter et al. 1995; Neutel et al. 2002, 2007) were also employed to demonstrate potential applications of the interaction strength. The diagonal strength (*S*) is the proportion of specific mortality caused by intraspecific competition. It is generally believed that the value range of *s* is between 0 and 1, and the system is considered to be stable; if *s* is greater than 1, the system is considered to be unstable. When comparing the stability of multiple systems, the smaller *s* is, the more stable the system (Neutel et al. 2002). Given the computational complexity of stability analysis indicators, an alternative empirical indicator was proposed to indicate changes in food web stability.

## 2 Materials and Methods

### 2.1 Study Site

**FIGURE 1:**
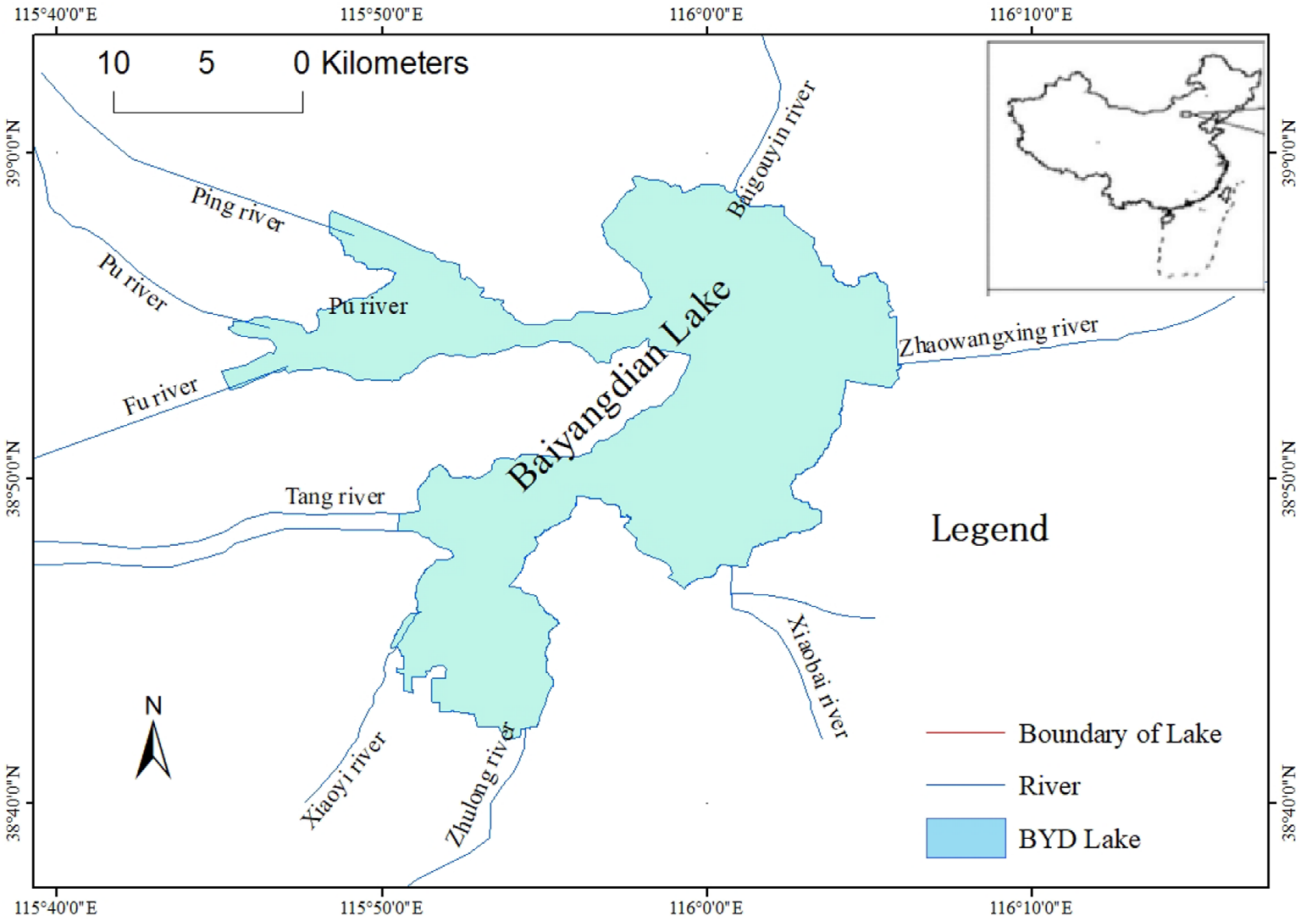
Location of the study area.

Lake Baiyangdian (BYD Lake) (38°44′ N to 38°59′ N, 115°45′ E to 116°07′ E) is located near the center of Hebei Province in China. It is the largest freshwater lake in northern China, covering an area of around 366 km^2^ with a mean depth of 2–3 m (Figure 1). The climate is dominated by a warm, temperate, and semi‐arid continental monsoon. The average annual temperature is 12.5°C (Liao et al. 2024), with the average annual precipitation being about 497 mm and the annual evaporation rate reaching 1637 mm/year (Cai et al. 2023).

Before 1960, BYD Lake was dominated by natural river input, but since then, dams and reservoirs have been constructed upstream for flood prevention, crop irrigation, and human consumption (Yang et al. 2022). Moreover, BYD Lake's watershed experienced severe drought, further shrinking the lake, which nearly dried up between 1984 and 1988. The water level decreased from 8.9 m in the 1950s to 6.9 m in the 2000s. At most monitoring sites, the water quality is classified as either class IV or V. Nevertheless, the required standard, the Environmental Quality Standards for Surface Water in China (GB 3838‐2002), for lakes is class III (Yang et al. 2019). As a typical macrophyte‐dominated shallow lake, BYD is under eutrophication pressure, and the dominant plant composition tends to shift from submerged species to floating‐leafed and emergent species (Han and Cui 2016). Since the 1990s, the species diversity of aquatic macrophytes, phytoplankton, zooplankton, macrobenthos, and fish in BYD Lake has continually decreased (Xu et al. 2013; Yi et al. 2020; Zeng et al. 2021).

### 2.2 Data

The bioenergetic parameters for each group for the Ecopath model of BYD Lake were derived from Guo et al. (2018, 2020) and Zeng et al. (2021). The Ecopath model is a mass‐balance ecosystem modeling framework designed to analyze trophic interactions and energy flows within aquatic and terrestrial ecosystems. It integrates biological, ecological, and fisheries data to quantify species biomass, consumption, production, and mortality rates within food webs. It has been widely applied in fisheries management and conservation, and ecosystem‐based management (Christensen and Walters 2004). Species composition of each functional group of the Ecopath model of the Baiyangdian ecosystem is shown in Table 1.

**TABLE 1:** Table species composition of each functional group of Ecopath model of Baiyangdian ecosystem.

Functional groups	Species
Detritus	Particles and dissolved organic matter
Macrophytes	*Ceratophyllum* L., *Charophyceae*, *Hydrilla verticillata* , *Myriophyllum verticillatum* L., *Najas marina* L., *Potamogeton crispus* L., *Vallisneria natans* (Lour.) Hara
Phytoplankton	*Cyanophyta* spp., *Cryptophyta* spp., *Pyrrophyta* spp., *Chrysophyta* spp., *Xanthophyta* spp., *Euglenophyta* spp., *Chlorophyt* spp.
Large zooplankton	*Cladocera* spp. and *Copepods* spp.
Meiofauna	*Chironomus*, *Gomphus*, *Sarcodina*, *Aulodrilus bretscher*, *Whitmania pigra* Whitman
Mollusk	*Anodonta*, *Bellamya*, *Cipangopaludina*, *Planorbis*, *Radix*, *Semisulcospira*
Herbivorous fish	*Ctenopharyngodon idellus*
Filter‐feeding fish	*Hypophthalmichthys molitrix* , *Hypophthalmichthys nobilis*
Fingerlings	*Abbottina rivularis* , *Hemiculter leucisculus* , *Pseudorasbora parva*
Small omnivorous fish	*Botia xanthi*, Juvenile *Carassius auratus* , *Pelteobagrus fulvidraco*
Omnivorous fish	*Cyprinus carpio*
Carnivorous fish	*Parasilurus asotus* , *Ophiocephalus argus*

Biomass data for each functional group from the 1950s to the 2020s were obtained from Yang et al. (2022), as listed in Table S1.

The dietary preferences of the consumer trophic groups of BYD Lake in historical representative years (i.e., 1958, 1980, 1993, 2009, and 2019) were gathered from Yang et al. (2022), as shown in Table S2. Apart from the dietary composition of carnivorous fish, the dietary composition of most functional species has remained mostly unchanged from the 1950s to the 2020s.

### 2.3 Calculating Interaction Strength Based on Ecopath's Parameters

According to de Ruiter et al. (1995), the dynamics of the trophic groups in a phytoplankton‐based food web can be described in terms of Lotka–Volterra‐type equations, such that

(1) $$\frac{{\mathrm{dX}}_{i}}{\mathrm{dt}}=X_{i}\left[r_{i}-\sum_{j=1}^{n}a_{\mathrm{ij}}X_{j}\right]$$

$X_{i}$, and $X_{j}$ represent the population size of groups *i* and *j*, respectively, $r_{i}$ is the specific rate of increase or decrease of group *i*, and $a_{\mathrm{ij}}$ is the coefficient of interaction between group *i* and group *j*. The coefficient of the interaction matrix describes the average direct effect that a single individual of one species has on a single individual of another species (i.e., a per capita effect, which differs from interaction strengths [a Jacobian community matrix]) (Laska and Wootton 1998). They have the following relationship:

(2) $$\alpha_{\mathrm{ij}}=a_{\mathrm{ij}}X^{*}$$

where $X^{*}$ is the vector of population densities when or near equilibrium.

By assuming $F_{\mathrm{ij}}^{*}=-a_{\mathrm{ij}}X_{i}^{*}X_{j}^{*}$, we can then calculate the interaction strengths between predator *j* and prey *i* (de Ruiter et al. 1995; Neutel et al. 2002):

(3) $$\alpha_{\mathrm{ij}}=\frac{-F_{\mathrm{ij}}^{*}}{X_{j}^{*}}$$

(4) $$\alpha_{\mathrm{ji}}=\frac{e_{j}F_{\mathrm{ij}}^{*}}{X_{i}^{*}}$$

where $\alpha_{\mathrm{ij}}$ denotes top‐down effects (negative interaction strengths), $\alpha_{\mathrm{ji}}$ denotes bottom‐up effects (positive interaction strengths), and $e_{j}$ is the efficiency with which food is converted into predator biomass.

The intraspecific interaction strength is assumed to be proportional to the natural death rate and is calculated as follows:

(5) $$\alpha_{\mathrm{ii}}=s\times d_{i},i=1,2,\cdots ,n$$

where $d_{i}$ refers to the natural specific death rate (per year) of species ($d_{i}<0$), and s (dimensionless) is the stability measure. We assume the same value of s for all trophic groups. The *s* is equal for each species for the simplicity of calculation. The magnitude of intraspecific interaction of species depends on the natural mortality rate of the species. The value of s leads to the minimum intraspecific interaction strength needed for matrix stability.

In Ecopath, the production‐biomass ratio (*P*/*B*) equals the instantaneous rate of total mortality (Christensen et al. 2005). Assuming that the organisms' annual average production balances the loss rate through natural death and predation ($P_{j}=d_{j}B_{j}+M_{j}$), the total consumption of the *j*th functional group ($F_{j}^{*}$) can be derived from Equation (6) (Bascompte et al. 2005). For taxa with more than one prey, $F_{j}^{*}$ is divided among prey items according to prey biomass ($B_{j}$) weighted by preference factors ($\omega_{\mathrm{ij}}$), as shown in Equation (7).

(6) $$F_{j}^{*}=\frac{d_{j}B_{j}+M_{j}}{e_{j}}=\frac{{\left(\frac{P}{B}\right)}_{j}\times B_{j}}{e_{j}}$$

(7) $$F_{\mathrm{ij}}^{*}=\frac{\omega_{\mathrm{ij}}B_{i}}{\sum_{k=1}^{n}\omega_{\mathrm{ij}}B_{k}}\times \frac{{\left(\frac{P}{B}\right)}_{j}\times B_{j}}{e_{j}}$$

where $F_{\mathrm{ij}}^{*}$ is the feeding rate per prey type, $B_{j}$ is the average annual population size, $M_{j}$ is the death rate due to predation, and $\omega_{\mathrm{ij}}$ is the preference of predator *j* for prey *i* over its other prey types. Calculations of predation rates begin with the top predators (that only die naturally) and proceed backward to the lowest trophic levels. All parameters can be obtained from the input and output of Ecopath model for the studied food web.

Detritus can be modeled following DeAngelis et al. (1989) and Moore et al. (1993), as revised Lotka–Volterra‐type equations expressing the dynamic process of the density of detritus ($X_{d}$):

(8) $$\frac{{\mathrm{dX}}_{d}}{\mathrm{dt}}=R_{d}+\sum_{i=1}^{n}\sum_{j=1}^{n}\left(1-{\mathrm{ac}}_{j}\right)a_{\mathrm{ij}}X_{i}X_{j}+\sum_{i=1}^{n}d_{i}X_{i}-\sum_{j=1}^{n}a_{\mathrm{dj}}X_{d}X_{j}$$

where $R_{d}$ is the input from an allochthonous source. Additionally, we can derive detritus cycles autochthonously as the unassimilated fractions of all prey killed, $\sum_{i=1}^{n}\sum_{j=1}^{n}\left(1-{\mathrm{ac}}_{j}\right)a_{\mathrm{ij}}X_{i}X_{j}$:

(9) $$\sum_{i=1}^{n}\sum_{j=1}^{n}a_{\mathrm{ij}}X_{i}X_{j}=\sum_{j=1}^{n}F_{j}^{*}=F_{T}^{*}$$

where $F_{T}^{*}$ is the total feeding rates of all predators, equals to the sum of the feeding rate of each predator ($F_{j}^{*}$). We assume that each predator has the same rate of unassimilation, ${\mathrm{ac}}_{j}$ = 0.2. Then, we have

(10) $$\sum_{i=1}^{n}\sum_{j=1}^{n}\left(1-{\mathrm{ac}}_{j}\right)a_{\mathrm{ij}}X_{i}X_{j}=0.8F_{T}^{*}$$

Next, $\sum_{i=1}^{n}d_{i}X_{i}$ represents the corpses of prey organisms that die from causes other than predation, which can be acquired from the table of mortality rates in the Ecopath model's output. Here, $d_{i}$ is other mortality besides predation for species *i* and $X_{i}$ is population density of *i*. The last item of the right side of Equation (8), $\sum_{j=1}^{n}a_{\mathrm{dj}}X_{d}X_{j}$ represents the total consumption of the detritus by detritivores. This detritus is consumed in a density‐dependent manner similar to the way organisms are consumed.

**FIGURE 2:**
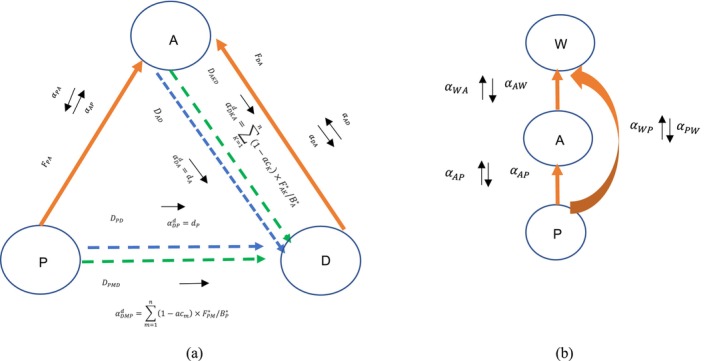
Omnivorous three‐species food web with and without considering detritus: Detritus food web (left) and phytoplankton‐based food web (right). The short black arrows indicate the direction of the interaction effect ( ). Red arrows indicate the feeding fluxes (F). The dashed arrows indicate the flux of dead organic matter (D). On the left, consumer group A feeds on the primary producer P and detritus D. On the right, group A feeds on the primary producer P and is prey for predator W.

However, Equation (8) only describes the total temporal change of detritus density. It is not specific to the material exchange between a particular functional group species and detritus. Therefore, a detailed analysis of all interaction processes between detrital and functional group species is needed that differs from the usual predator–prey food web. The interspecies interactions in the detrital and phytoplankton‐based food webs are shown in Figure 2, respectively.

In the case on the left in Figure 2a, the detrital food web consists of a consumer (*A*) feeding on the primary producer (*P*) and detritus (*D*). There are three types of interactions and corresponding calculation methods. First, a feeding flux ($F_{\mathrm{PA}}$) from *P* to *A* corresponds to a pair of interspecies interactions, namely the negative top‐down predation effect ($\alpha_{\mathrm{PA}}$) and the positive bottom‐up prey effect ($\alpha_{\mathrm{AP}}$). This first type of interaction (i.e., between *A* and *P*) is equivalent to a predator–prey interaction and can be calculated using the method of de Ruiter et al. (1995) based on Equations (3) and (4). The second type interaction is that from *P* to *D*, where another two dead organic matter flux ($D_{\mathrm{PD}}$ and $D_{\mathrm{PMD}}$) decides an aggregate positive interspecies interaction ($\alpha_{\mathrm{DP}}^{\tau }$) from *P* to *D*, as shown in Figure 2a:

(11) $$\alpha_{\mathrm{DP}}^{\tau }=\alpha_{\mathrm{DMP}}^{d}+\alpha_{\mathrm{DP}}^{d}=\sum_{m=1}^{n}\left(1-{\mathrm{ac}}_{m}\right)\times F_{\mathrm{PM}}^{*}/B_{P}^{*}+d_{P}$$

where $\alpha_{\mathrm{DMP}}^{d}$ is the interaction of *P* to *D* from *P* as unassimilated food of other predators (*M*, including *A*), $\alpha_{\mathrm{DP}}^{d}$ is interaction of *P* to *D* from the mortality of *P* from causes other than predation, ${\mathrm{ac}}_{m}$ is the unassimilation rate of *M*, where ${\mathrm{ac}}_{m}$ = 0.2, $F_{\mathrm{PM}}^{*}$ is the equilibrium feed rate of predator *M* to prey *P*, $B_{P}^{*}$ is the equilibrium population size of species *P*, and $d_{P}$ is the non‐predation mortality of species *P*.

The most complex interaction is the third interaction type between *A* and *D*. The complexity is manifested by the inclusion of three material fluxes: one feeding flux ($F_{\mathrm{DA}}$) and two dead organic matter fluxes ($D_{\mathrm{AD}}$ and $D_{\mathrm{AKD}}$). Furthermore, it involves other predators that feed on *A*, for example, species *k*. To our knowledge, this complexity has been overlooked in previous literature. As shown in Figure 2a, four interspecies interactions should be aggregated to two elements of the interaction strength to meet its definitions. According to the direction of the arrow in the figure, one positive bottom‐up prey effect ($\alpha_{\mathrm{AD}}$) can be calculated as the same way as predator–prey interactions. The other three interactions ($\alpha_{\mathrm{DA}}$, $\alpha_{\mathrm{DKA}}^{d}$, and $\alpha_{\mathrm{DA}}^{d}$) from *A* to *D* should be aggregated as the integrated interaction strength $\alpha_{\mathrm{DA}}^{\tau }$. In other words, $\alpha_{\mathrm{DA}}^{\tau }$ consists of three parts. The first part is a negative interaction from predator *A* to pre*y D* ($\alpha_{\mathrm{DA}}$) besides predation. The second part is a positive interaction from the non‐predation mortality of *A* ($\alpha_{\mathrm{DA}}^{d}$). The third part is a positive interaction from the unassimilated food of other predators ($\alpha_{\mathrm{DKA}}^{d}$):

(12) $$\alpha_{\mathrm{DA}}^{\tau }=\alpha_{\mathrm{DA}}+\alpha_{\mathrm{DA}}^{d}+\alpha_{\mathrm{DKA}}^{d}=\frac{-F_{\mathrm{DA}}^{*}}{B_{A}^{*}}+d_{A}+\sum_{K=1}^{n}\left(1-{\mathrm{ac}}_{K}\right)\times F_{\mathrm{AK}}^{*}/B_{A}^{*}$$

where $F_{\mathrm{DA}}^{*}$ is the equilibrium feed rate of predator *A* to prey *D*, $B_{A}^{*}$ is equilibrium population size of species *A*, $d_{A}$ is the non‐predation mortality of species *A*, ${\mathrm{ac}}_{K}$ is the unassimilation rate, where ${\mathrm{ac}}_{K}$ = 0.2, and $F_{\mathrm{AK}}^{*}$ the equilibrium feed rate of predator *K* to prey *A*, where k=1ton.

The interactions in the grazing food web are shown in Figure 2b. In a common predator–prey food web, a pair of interaction strengths exists with every feed flux, namely, a top‐to‐bottom and a bottom‐to‐top interaction, calculated according to Equations (3) and (4), respectively.

After calculating the interaction strength considering the energy cycle of detritus, the theory of the minimum diagonal strength (*S*) needed for matrix stability is employed to carry out stability analysis (Neutel et al. 2002). The diagonal refers to the elements along the main diagonal of the community matrix. It represents the magnitude of the $a_{\mathrm{ii}}$ terms, which are the intra‐specific interaction strength terms. It stabilizes food webs by reinforcing self‐limiting mechanisms. The computational procedure for determining *S* is given in the supporting information of Neutel et al. (2007). A matrix was first arbitrarily assigned an initial *S* value (e.g., *S* = 1). Then, we decided whether the system was stable using May's stability analysis method (May 1972). May's stability analysis constructs the Jacobian (Community) matrix for the differential equations of species dynamics (with the intra‐species interactions on the main diagonal and the inter‐species interactions on the off‐diagonal), and calculates the maximum real part of its eigenvalues: if it is negative, the system is locally stable. If the value of the real part of the largest eigenvalue of the matrix was not close enough to zero, trial‐and‐error method with an order of magnitude step was carried out to obtain *S* until it is sufficiently accurate.

### 2.4 Loop Weight Analysis

Neutel et al. (2002) found that the maximum of all the loop weights in community matrix A is an approximation of the value *S* for matrix stability:

(13) $$S=\max_{k,E_{k}}\left({\left|\alpha_{i_{1}i_{2}}\cdot \alpha_{i_{2}i_{3}}\cdot \cdots \cdot \alpha_{i_{k}i_{1}}\;\right|}^{1/k}\right)$$

where $E_{k}$ is the set of loops of length *k*. Thus, we used the maximum loop weight (MLW) as an approximation of the level of intraspecific interaction sufficient for stability, and designated matrices with a smaller *S* as “more stable.”

However, the total number of feedback loops in a food web was large, making it tedious to calculate the weight of each loop. Therefore, we employed a simplified version of stability indicator *S*. Loops with different lengths play different roles in determining the stability of a food web, and the loop weights decrease with the loop lengths. Loop length refers to the number of links (or species) in a closed trophic chain. However, the shortest feedback loops, the predator–prey loops of two links (generating negative feedback), do not contribute to instability in these systems (Neutel et al. 2007). The strongest omnivorous feedback, a three‐link positive feedback, is a good indicator of system stability (Mitchell and Neute 2012). In summary, the maximum weight of omnivorous loops in all food webs was dominated by length three anticlockwise loops, which started with the omnivores with two top‐down effects and one bottom‐up effect. Therefore, the maximum loop weight of a food web was derived by comparing and calculating all length three loop weights.

## 3 Results

**FIGURE 3:**
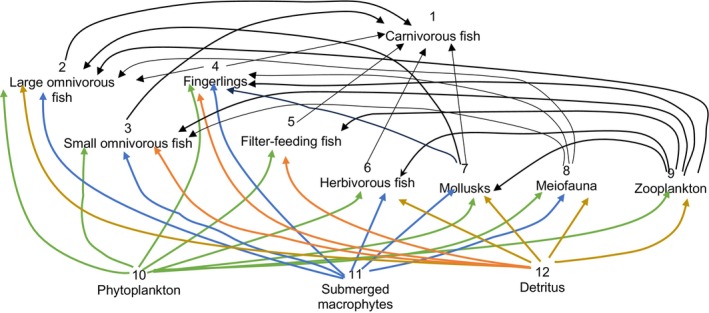
Food web relationships and trophic levels in BYD Lake. (1) Green, blue, and orange lines represent the starting points of the food chain, which are phytoplankton, submerged macrophytes, and detritus, respectively. (2) From bottom to top, the position of species reflects their trophic levels (1‐3.427) from low to high. (3) The flow to the detritus is not represented here but was included in the analyses.

The food web relationships and trophic levels in BYD Lake are shown in Figure 3.

Table S3 shows the estimated outputs from the Ecopath model of BYD Lake from the 1950s to 2020s. Table S4 shows the calculated results of the Jacobian matrix of detritus and phytoplankton‐based food webs of BYD Lake from the 1950s to the 2020s. The main difference between the Jacobian matrix of detritus food webs and phytoplankton‐based food webs was the interaction strengths between detritus and other functional group species, which was calculated without considering the detritus formed from dying individuals and formed by unassimilated food (feces) in the latter. The interaction strength of species to detritus depended on the positive contribution of the unassimilated food of other species, in addition to their natural specific death, including all non‐predatory losses, as well as loss due to feeding on detritus. When the value of the first two items (the unassimilated fractions and the non‐predation mortality of species) was more significant than that of the last item (the total consumption of the detritus by detritivores), the signs of the interaction strength of species to detritus were positive, such as those of CarF, HerF, Phyt, and SubM shown in Table S4. Conversely, the signs were negative, such as those of LomF, SomF, Fing, FilF, Moll, Meio, and Zoop, whose predator–prey effect dominated other interactions between species and detritus. However, only the prey on detritus was considered in phytoplankton‐based food webs, for example, zooplankton, meiofauna, mollusks, filter‐feeding fish, fingerlings, omnivorous fish. They are all partially and wholly feeding on detritus. The other two types of interactions were not included, resulting in calculated results that were smaller than those for the detritus food web. For example, in the detritus food web and the phytoplankton‐based food web, the interaction between the species that mainly feed on detritus and detritus is not much different, indicating that in the role of predation and sink (the unassimilated fractions and the non‐predation mortality of species), predation has a greater impact on the interaction.

**FIGURE 4:**
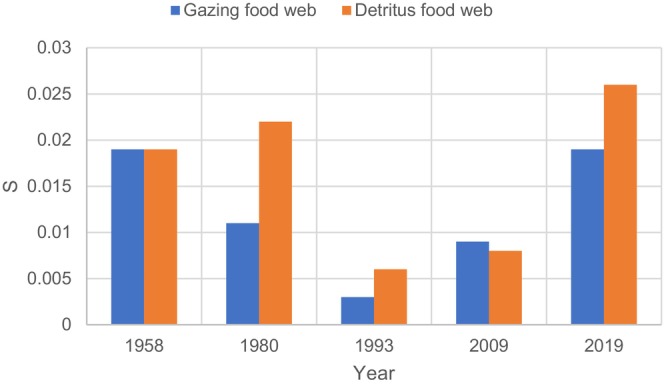
Stability results of two types of food webs in BYD Lake.

The calculating results of *S* for two types of food webs (namely, the detritus food web and phytoplankton‐based food web) are shown in Figure 4.

The energy flow of detritus has a significant impact on the estimated interaction strength. The diagonal strength between the detritus food web and phytoplankton‐based food web showed little difference in 1958. The absolute values of relative deviations in the other years ranged from 12.5% to 50%, averaging 34.9%. This indicated obvious differences in the stability trends of two types of food webs throughout time. For example, stability trends first rose and then fell (*S* values first decreased and then increased) in the phytoplankton‐based food web. However, stability first decreased, rose, and then fell (*S* values first increased, then decreased, and then increased) in the detritus food web.

All length three loop weights of the BYD Lake food web are shown in Table 2.

**TABLE 2:** The Loop‐weight of the feedback loop (loop length = 3) under different years.

Serial number	Feedback loops	Years				
		1958	1980	1993	2009	2019
1	Phyt > Fing > LomF	0.0033	0.0025	0.0035	0.0221	0.0133
2	Phyt > Moll > Fing	0.0359	0.0154	0.0156	0.0382	0.0291
3	Phyt > Moll > LomF	0.0144	0.0084	0.0071	0.0490	0.0286
4	Phyt > Meao > Fing	0.0073	0.0066	0.0127	0.0358	0.0077
5	Phyt > Meao > LomF	0.0021	0.0026	0.0045	0.0243	0.0068
6	Phyt > Meao > SomF	0.0012	0.0014	0.0023	0.0120	0.0094
7	Phyt > Zoop > LomF	0.0052	0.0080	0.0146	0.0650	0.0873
8	Phyt > Zoop > Fing	0.0594	0.0415	0.0603	0.1022	0.0876
9	Phyt > Zoop > SomF	0.0080	0.0133	0.0073	0.0316	0.1051
10	Phyt > Zoop > FilF	0.2062	0.2246	0.2685	0.3818	0.0940
11	Phyt > Zoop > HerF	0.0314	0.0011	0	0	0.0842
12	Phyt > Zoop > Moll	0.0139	0.0214	0.0170	0.0261	0.1254
13	SubM > Fing > LomF	0.0641	0.0457	0.0582	0.0117	0.1139
14	SubM > Moll > Fing	0.2109	0.0980	0.0804	0.0480	0.1964
15	SubM > Moll > LomF	0.2253	0.1076	0.0915	0.0718	0.1429
16	SubM > Meao > Fing	0.0207	0.0219	0.0324	0.0366	0.0632
17	SubM > Meao > LomF	0.0156	0.0170	0.0278	0.0289	0.0414
18	SubM > Meao > SomF	0.0134	0.0132	0.0215	0.0180	0.0806
19	Detr > Fing > LomF	0.0553	0.0255	0.0446	0.0550	0.0765
20	Detr > Fing > Phyt	0.0488	0.0810	0.0421	0.1156	0.0399
21	Detr > Fing > SubM	0.4025	0.3613	0.3150	0.3412	0.2912
22	Detr > FilF > Phyt	0.1233	0.0942	0.0843	0.1949	0.0504
23	Detr > HerF > Phyt	0.0086	0	0	0.0154	0.0427
24	Detr > HerF > SubM	0.1380	0	0	0.2167	0.2335
25	Detr > Moll > Fing	0.3365	0.3992	0.1322	0.1493	0.2716
26	Detr > Moll > LomF	0.3272	0.1339	0.1405	0.2017	0.2833
27	Detr > Moll > Phyt	0.1061	0.1633	0.0527	0.1106	0.1451
**28**	**Detr > Moll > SubM**	**0.7106**	**0.4905**	**0.3085**	**0.1847**	**0.6170**
29	Detr > Meio > Fing	0.0723	0.0620	0.1207	0.1771	0.0702
30	Detr > Meio > LomF	0.0497	0.0454	0.0996	0.1266	0.0662
31	Detr > Meio > SomF	0.0330	0.0324	0.0695	0.0885	0.0817
32	Detr > Meio > Phyt	0.0684	0.0684	0.0906	0.1228	0.0370
33	Detr > Meio > SubM	0.2222	0.2222	0.2528	0.1668	0.1916
34	Detr > Zoop > LomF	0.1127	0.1300	0.2940	0.3093	0.3041
35	Detr > Zoop > SomF	0.1880	0.1231	0.1951	0.2137	0.3285
**36**	**Detr > Zoop > Fing**	**0.5250**	**0.3566**	**0.5175**	**0.4611**	**0.2851**
**37**	**Detr > Zoop > FilF**	**0.7729**	**0.7295**	**0.6954**	**0.7113**	**0.3556**
38	Detr > Zoop > HerF	0.0303	0.0104	0	0	0.2274
**39**	**Detr > Zoop > Moll**	**0.3903**	**0.2825**	**0.2273**	**0.1946**	**0.7620**
**40**	**Detr > Zoop > Phyt**	**0.3052**	**0.3052**	**0.2909**	**0.4074**	**0.7896**
41	Max. loop weight	**0.7729**	**0.7295**	**0.6954**	**0.7113**	**0.7896**
42	Required *s*	0.019	0.022	0.006	0.008	0.026

*Note:* Text in bold indicates a heavier loop.

Abbreviations: CarF, carnivorous fish; Detr, detritus; FilF, filter‐feeding fish; Fing, fingerlings; HerF, herbivorous fish; LomF, large omnivorous fish; Meio, meiofauna; MolL, mollusks; PhyT, phytoplankton; SomF, small omnivorous fish; SubM, submerged macrophytes; ZooP, zooplankton.

There were 40 kinds of length three loops in the BYD Lake detritus food web. Twelve loops started from phytoplankton, six started from submerged macrophytes, and up to 22 started from detritus. The average loop weights starting from phytoplankton, submerged macrophytes, and detritus were 0.044, 0.067, and 0.211, respectively. These results showed that the detritus‐based loop weights were heavier than the other two categories, governing the stability of the food web. The *MLW* differed in different periods, changing from detritus > zooplankton > filter‐feeding fish (1958–2009) to detritus > zooplankton > phytoplankton (2019) and implying that the food web structure underwent significant changes.

**FIGURE 5:**
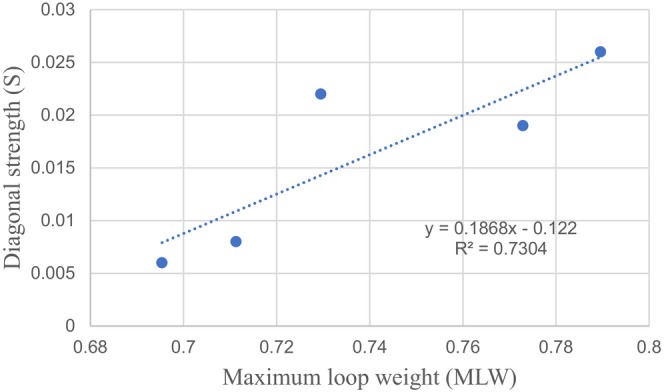
Relationship between stability (*S*) and maximum loop weight (*MLW*).

The relationship between the *S* and *MLW* in BYD Lake food web is shown in Figure 5.

A highly significant positive correlation between *S* and *MLW* was found (*R* ^2^ = 0.73). The value of *R* ^2^ ranged between 0.71 and 0.94 (Neutel et al. 2007). The relatively low correlation of our results may be due to the poor quality of long‐time series data, especially on account of the many uncertainties in the early stages. According to Neutel et al. (2007), the observed strong relationship between stability and the maximum weight of omnivorous loops was robust and did not depend on the specific food web types and configurations, or on the range of complexity observed in these webs.

## 4 Discussion

The elements of interaction strengths are well defined in theory. However, there are few empirical estimates of these interaction strengths (de Ruiter et al. 1995). This may be because related parameters are rarely acquired from the field or in lab experiments (Landi et al. 2018). After de Ruiter et al. (1995) proposed energetics methods based on direct observations of the material flow of food webs, the interaction strength can be estimated following standard Lotka–Volterra equations. The parameters needed during such calculations include feeding rates, observed population sizes, death rates, and energy conversion efficiencies. However, it is not easy to obtain the energy parameters of a food web directly from monitoring or by reference to previous literature. In order to fill the gaps, the Ecopath model, as a vehicle to infer difficult‐to‐measure trophic exchange parameters, was introduced to calculate interaction strengths. Almost all parameters required in the calculation process were obtained from the Ecopath model, which greatly facilitated calculations of the interaction strength (Tables S1 and S2).

Detritus and phytoplankton are the two major food sources for primary consumers in lakes (Safi et al. 2019). According to network analysis metrics, a detritus‐based food web is more mature, stable, and resilient than a phytoplankton‐based food web (Zeng et al. 2021). The feedback loop of detritus–phytoplankton–zooplankton constitutes the most critical loop in most lake food webs for highest loop weight and significant destabilizing effects (Kuiper et al. 2015). Moreover, the results of this study also confirmed the considerable differences between detritus and phytoplankton‐based food webs. Therefore, the full interactions between detritus and related species could not be ignored when analyzing the stability of lake food webs. Many interactions within ecosystems have negligible effects on community dynamics, with only a few key trophic interactions playing a dominant role in the deterioration of food web stability (Kuiper et al. 2015; Jacquet et al. 2016; Zhang, Yi, et al. 2023). In real food webs, interaction strengths are organized in trophic loops in such a way that long loops contain relatively many weak links (Neutel et al. 2002). The loop with highest weight is crucial for the system's stability (Neutel et al. 2007). Quantifying the highest weight of the loop could be a useful tool for exploring the stability of aquatic ecosystems. Few case studies show that the zooplankton, diatoms, and detritus loop become the highest weight loop (Kuiper et al. 2015; Zhang, Yi, et al. 2023). In this loop, detritus (non‐living organic material) serves as a nutrient source for phytoplankton, which in turn supports zooplankton populations that graze on the phytoplankton (Edwards 2001; Perhar and Arhonditsis 2012). It may reflect the dominant bottom‐up control within the system (Li et al. 2014; Frau et al. 2019). In eutrophic systems, bottom‐up control can overpower other mechanisms like grazing pressure from zooplankton or predation from fish, particularly when nutrients are abundant (Matsuzaki et al. 2018). In our study, the primary dominant loop in most periods was the detritus > zooplankton > filter‐feeding fish. It infers dominant top‐down control within the system (Lin et al. 2021). This top‐down control occurs because the filter‐feeding fish can reduce zooplankton populations through direct grazing pressure, which, in turn, may lead to changes in the dynamics of detritus and phytoplankton, as zooplankton are key in regulating primary producers like phytoplankton (Shan et al. 2014; Zhang, Mei, et al. 2023). Recent studies have also shown that food web stability metrics can serve as an indicator of ecosystem state transitions. By monitoring changes in these critical loops, it is possible to better predict ecological changes in lakes and take timely management measures to maintain ecological balance (Ma et al. 2023).

Although some literature (Hunt et al. 1987; DeAngelis et al. 1989; Moore et al. 1993, 2004; de Ruiter et al. 1995) has suggested considering the material flow between species and detritus when studying the energetic flow characteristics of food webs, these studies have not fully demonstrated how interaction strengths should be calculated. Unlike the interaction strength of a typical predator–prey food web, a detritus food web has more complex processes between individual species and detritus, as shown in Section 2.2. The complexities are exacerbated by the three types of interaction strengths, from the most straightforward predator–prey interaction to the most complicated processes, including that of the unassimilated food of predators (accumulated for multiple predators), that of species natural mortality, and that of predator–prey interactions.

In the particular case of BYD Lake, three studies (Yang et al. 2022; Zhang et al. 2022; Zhang, Yi, et al. 2023) analyzing its stability did not fully consider the interactions between functional group species and detritus. Yang et al. (2022) focused only on interactions between species and did not consider any interaction between detritus and other species. The studies by Zhang et al. (2022); Zhang, Yi, et al. (2023) considered only the simplest predator–prey relationship between individual species and detritus, neglecting the other interactions. The results of these studies showed that the stability of BYD Lake first increased and then decreased from the 1950s to 2019. This indicates that the ecosystem of BYD Lake began as a mature and stable system from the 1950s to the 1990s and then moved toward instability after the 1990s (Zhang et al. 2022). This trend agrees well with our results regarding the phytoplankton‐based food web. However, previous studies failed to identify the trend of the detrital food web of BYD Lake, in which the ecosystem of BYD Lake moved toward an unstable state from the 1950s to 1980s. Mao et al. (2024) identified two ecological shifts based on diatom assemblages from an analysis of a sediment core. The first ecological shift occurred around the year 1963 and was associated with the rather abrupt physical changes, that is, damming in the basin. The second ecological shift occurred in the 1990s and was attributed to sustained nutrient loading due to the intensification of human activity as well as an increase in the regional temperature. These results agree well with the two unstable moving periods identified in our study (the stability trend of the detritus food web).

**FIGURE 6:**
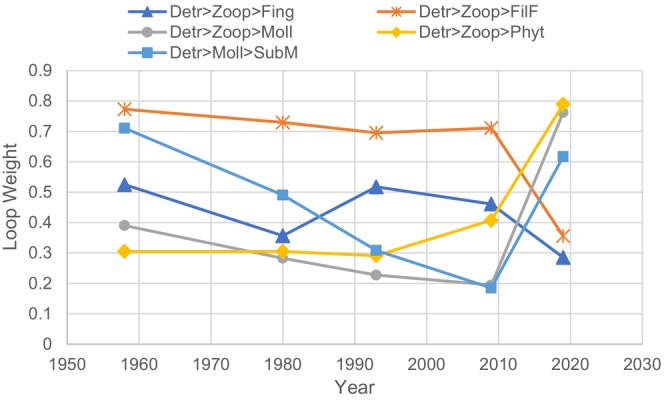
Variation of the maximum loop weight over time. Fing, fingerlings; FilF, filter‐feeding fish; MolL, mollusks; ZooP, zooplankton; Phyt, phytoplankton; SubM, submerged macrophytes; Detr, detritus.

It is well‐known that the interaction strength and patterns significantly impact the stability of a food web (de Ruiter et al. 1995; Bascompte et al. 2005; Neutel et al. 2007). To identify the consequences of the interactions of crucial species in a food web, loop weight analysis (LWA) can be used to explore the structure and organization of complex communities. LWA can also be used to identify the most critical loop that affects food web stability, and to explain the impact of species manipulation on food web stability (Neutel et al. 2002). Multiple factors, such as organism traits and biomass structure (Neutel et al. 2007), influence maximum loop weight (*MLW*), which determines food web stability. As a result of biomass variation, *MLW* may change over time in terms of both the weight value and the composition of the loop (de Ruiter et al. 1995; Neutel et al. 2007). Another reason for the variation of *MLW* is that the energetic data used to infer food web structure are not necessarily accurate, and there is considerable uncertainty when quantifying *MLW*. Therefore, it is essential to focus on a few feedback loops, rather than the one having the maximum loop weight. For example, most studies (Neutel et al. 2007; Kuiper et al. 2015) focused on only one loop with the *MLW*. However, our results showed that the loop structure contributing to *MLW* was constant from 1950 to 2009 but changed in 2019, as shown in Figure 6.

Therefore, it is necessary to examine heavier loops that may have a chance of evolving into the heaviest ones when catastrophic and consequential changes to the food web structure occur, as shown in Figure 6. Note that the degree of stability change was positively correlated with the weight changes of the heavier loops.

**FIGURE 7:**
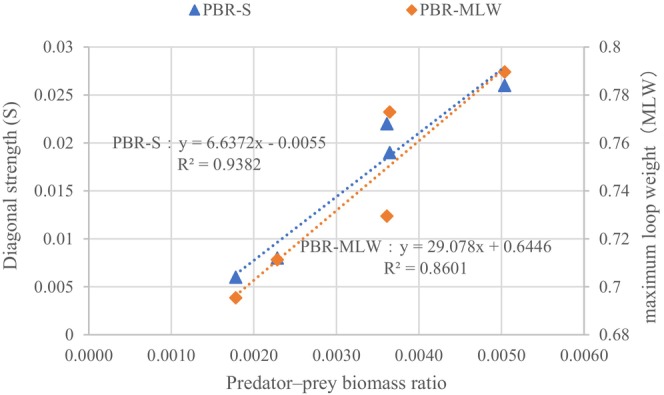
Correlation between predator–prey biomass ratio (*PBR*) and diagonal strength (*S*) and maximum loop weight (*MLW*).

The impact of species dynamics on food web stability can be analyzed using the predator–prey biomass ratios in these omnivorous loops. These ratios were shown to have a crucial role in preserving stability as productivity and complexity increased during succession (Neutel et al. 2007). The correlations between the predator–prey biomass ratios and diagonal strength (*S*), and between the predator–prey biomass ratios and *MLW*, are shown in Figure 7.

**FIGURE 8:**
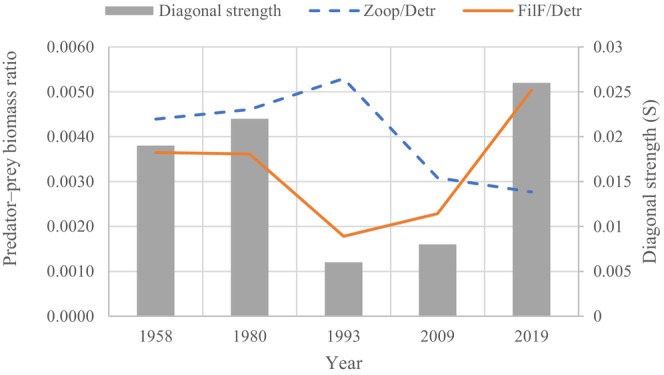
Trend of diagonal strength and predator–prey biomass ratio from the 1950s to the 2020s. The red solid line represents the predator‐prey biomass ratio (filter‐feeding fish and detritus). The blue dashed line represents the predator‐prey biomass ratio (zooplankton and detritus). The gray columns represent the corresponding diagonal strength at different times.

The predator–prey biomass ratio was strongly related to food web stability and determined the maximum loop weight of a three‐link omnivorous loop. Low top‐bottom biomass ratios contributed to less loop weight and increased stability. The diagonal strength and predator–prey biomass ratio trends from the 1950s to 2019 are shown in Figure 8.

The trend of the predator–prey biomass ratio (filter‐feeding fish and detritus) was almost consistent with the trend of diagonal strength. This showed that the trend of stability first decreased slightly, then increased, and finally decreased significantly. From 1958 to 2009, the stability of the food web was limited by a three‐link omnivorous loop: detritus > zooplankton > filter‐feeding fish. As the biomass ratio (filter‐feeding fish/detritus) increased, the instability of the food web increased, and vice versa. However, the new loop (detritus > zooplankton > phytoplankton) higher in the web became the heaviest, and new biomass ratios (zooplankton/detritus) determined stability, as shown from 2009 to 2019. Our analyses revealed that only a few trophic interactions dictated the deterioration of food web stability, particularly among the omnivorous loop of detritus > zooplankton > filter‐feeding fish, or among the omnivorous loop of detritus > zooplankton > phytoplankton.

Food web stability can be used as an empirical indicator of ecosystem resilience (Kuiper et al. 2015). It signals the state transition in a real lake ecosystem (Zhang, Yi, et al. 2023). Many researchers have focused on realizing early warnings of lake ecosystem state transitions and revealing the process and mechanism by which nutrients affect ecosystem stability (Kong et al. 2017; Zhang et al. 2021).

**FIGURE 9:**
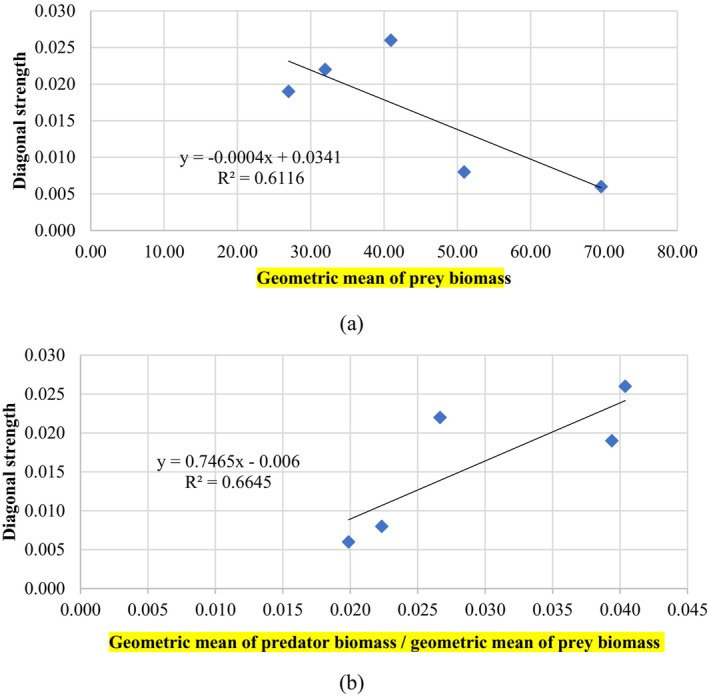
Correlation between the empirical indexes and diagonal strength (*S*).

There are two categories of methods of measuring food web stability: theoretical studies for the asymptotic stability of the equilibrium based on a community matrix, and temporal stability in studies of empirical time series (Landi et al. 2018). For example, for theoretical methods, diagonal strengths and *MLW* require an estimate of the interaction strength matrix typically derived through some modeling process. By contrast, empirical studies on stability definitions are based on a system's ability to defy change. The variable nature of population dynamics, found in both field and laboratory experiments, has led experimentalists to use measures of variability as indices of a system's stability (McCann 2000), such as the coefficient of variation of biomass (Eschenbrenner and Thébault 2023), and resistance (Pennekamp et al. 2018). Empirical indicators are easier to acquire, but they are only descriptive and non‐explanatory, whereas theoretical indicators are the opposite. To facilitate the use of stability indicators in ecological management practice, it is necessary to find the connections between the two types of indicators and further construct practical, easily accessible empirical indicators. Here, we propose a geometric mean ratio between predator and prey biomass as an alternative for *S* or *MLW*, which is as follows:

(14) $${\left(\frac{B_{C}}{B_{P}}\right)}_{t}=\frac{\sqrt[{k}]{B_{C1,t}\times B_{C2,t}\times \cdots \times B_{\mathrm{Ck},t}}}{\sqrt[{k}]{B_{P1,t}\times B_{P2,t}\times \cdots \times B_{\mathrm{Pk},t}}}$$

where ${\left(\frac{B_{C}}{B_{P}}\right)}_{t}$ is the geometric mean ratio between the predator and prey biomass in period *t*. The reason for selecting the indicator lies in a few food web theories showing that top‐down control plays a key role in community assembly. The shape of the biomass pyramid indicates that more biomass at the top of food chains limits stability (Neutel et al. 2007). Lake stability can be influenced by bottom‐up or top‐down interactions depending on ecological conditions. In eutrophic lakes, where nutrient availability drives primary productivity, bottom‐up control is dominant, leading to algal blooms and ecosystem destabilization (Li et al. 2020). Stability is also driven by bottom‐up effects in lakes with low predator density, where resource supply directly impacts ecosystem dynamics (Rejas et al. 2005). In mesotrophic lakes, top‐down control prevails, where predators regulate zooplankton populations, indirectly affecting primary producers (Carpenter et al. 2001). Reynolds (1994) argues that cascades are most likely to control phytoplankton in small, shallow, unstratified lakes capable of supporting extensive macrophytes. Bess et al. (2021) found that the predatory behavior of zooplankton has an important impact on the stability of the lake ecosystem. However, in marine food webs, bottom‐up and top‐down forces can interact in a non‐linear manner, influencing the system's overall dynamics (Lynam et al. 2017). Before 2009, the loop with the heaviest loop weight is detritus > zooplankton > filter‐feeding fish, which indicates that the top‐down interaction plays a crucial role in the stability of the food web. In 2019, the loop with the heaviest loop weight changes to detritus > zooplankton > phytoplankton, which implies that the bottom‐up interaction plays an important role. Despite some controversy, a wide range of research across multiple taxa has established that carnivores strongly influence prey population dynamics through both direct biomass reduction and indirect risk effects (Winnie and Creel 2017). Because of these powerful top‐down effects, carnivores can influence ecosystems across multiple trophic levels, as well as the overall ecosystem structure and function (Winnie and Creel 2017). Therefore, the selected indicator essentially represents top‐down effects, where the larger the value, the weaker the stability. The connections between ${\left(\frac{B_{C}}{B_{P}}\right)}_{t}$ and other biomass‐type indicators and diagonal stability ($S_{t}$) are shown in Figure 9.

Compared with other biomass‐related indicators, the correlation between the geometric mean of total biomass of the system (including detritus) and predator biomass and the diagonal strength is very poor, and the *R* ^2^ is 0.0682 and 0.0039 respectively. The correlation between ${\left(\frac{B_{C}}{B_{P}}\right)}_{t}$ and the diagonal strength ($S_{t}$) was the most significant (*R* ^2^ = 0.6645, *p* < 0.08). The most important feature of this indicator was that it avoided calculating complex structural information such as the material flow, interaction, diversity, patterns, and feeding rate between species. Moreover, this indicator was more accessible to obtain and was closely related to diagonal strength, thus, it can be used as an alternative indicator in management practice.

Previous studies demonstrated that food web stability is affected by multiple disturbances that lead to changes in the system state. To maintain a good state of the lake, three perspectives, hydrological management, water quality restoration, and biological manipulation, were suggested to maintain food web stability (Zhang et al. 2022). Finally, the food web stability was adjusted by bio‐manipulation based on our loop weight analysis. For example, it was necessary to manipulate filter‐feeding fish below the density threshold (Kong et al. 2016) because they have a significant negative impact on zooplankton, thereby weakening the grazing effect of zooplankton on detritus. In addition, long‐term monitoring of all species types, biomass, and feeding habits is still necessary when conducting theoretical analysis, such as the analysis of *S* and *MLW*, or at least monitoring the biomass of species when empirical analysis is employed, such as ${\left(\frac{B_{C}}{B_{P}}\right)}_{t}$.

## 5 Conclusion

Accurate measurements of the interaction strengths between functional groups remain an ongoing problem in ecology, especially for the diagonal elements describing intraspecific interactions. Starting from a generalized form of the Lotka–Volterra equations for phytoplankton‐based food webs, we derived a method of measuring the elements of the Jacobian matrix (i.e., the interaction strength) based on the characteristic energy parameters provided by Ecopath. The total interaction between functional groups and detritus was thus modeled by following these revised Lotka–Volterra equations, including the interaction between functional groups and detritus. The interactions between living groups and detritus were the production of detritus as unassimilated food, the production of detritus as non‐predation mortality, and the consumption of detritus as food for living groups. We proposed a detailed procedure for measuring elements of the interaction strength of a detritus‐based food web based on output parameters of the Ecopath model. To our knowledge, this has not been illustrated clearly in previous studies.

We took the food web of BYD Lake from 1958 to 2019 as an example. The results showed considerable differences in the Jacobian matrix before and after considering detritus, with average relative deviations of 34.9%. Similar previous results agreed well with the two unstable moving periods identified by our study of stability trends of the detritus food web of BYD Lake.

Based on the real interaction strength, we used loop weight analysis to explore the structure and organization of complex communities. We also identified the most critical loop that affected food web stability and explained the impact of species manipulation on food web stability. From 1958 to 2009, the stability of BYD Lake was limited by a three‐link omnivorous loop: Detritus > zooplankton > filter‐feeding fish. As the predator–prey biomass ratio (filter‐feeding fish/detritus) increased, instability increased, and vice versa. However, the new loop (detritus > zooplankton > phytoplankton) and corresponding new predator–prey biomass ratios (zooplankton/detritus) resulted in stability from 2009 to 2019.

Although loop weight analysis is useful, it is computationally complex, and high‐quality data are required. A geometric mean ratio of predator biomass to prey biomass was thus proposed to connect theoretical indexes such as the diagonal strength and maximum loop weight for easier accessibility in ecosystem management practices. The regression model showed a positive correlation (*R* ^2^ = 0.6645, *p* < 0.08). We recommend using this approach as an early warning indicator for ecological stability management.

This study re‐examined food web stability considering detritus flow. We demonstrated its importance and potential use in management practice. The proposed empirical indicator can act as a precautionary indicator, though it is not precise. It is an attempt to connect the theoretical stability indicator and descriptive metrics to inform management strategy and action. Future research directions to connect theoretical and empirical indicators include further verifying the reliability of the relationship between the theoretical and empirical indicators and finding more empirical indicators that precisely indicate changes in stability. Further effort is needed to develop the conditions for applying theoretical stability indicators in ecological management practice, such as long‐term comprehensive monitoring of species dynamics and energy flow, developing calculation or analysis software, constructing a database of the energetic characteristics of species, and more experience in stability management and regulation.

## Author Contributions

**Yong Zeng:** conceptualization (lead), methodology (lead), writing – original draft (lead), writing – review and editing (lead). **Yanwei Zhao:** data curation (equal), formal analysis (equal), funding acquisition (equal). **Wei Yang:** conceptualization (supporting), data curation (equal), formal analysis (equal), funding acquisition (equal).

## Conflicts of Interest

The authors declare no conflicts of interest.

## Supporting information


**Appendix S1:** ece371934‐sup‐0001‐AppendixS1.docx.

## Data Availability

The data presented in this study are available in this manuscript.
