# Supplementary material for: New Approaches to Assess Food Web Stability in Aquatic Ecosystems: A Case Study on Baiyangdian Lake
Source: Ecol Evol. 2025 Aug 8;15(8):e71934. doi: 10.1002/ece3.71934 (PMC12334362; doi:10.1002/ece3.71934)
Supplement: Supplementary file 1 — Appendix S1: ece371934‐sup‐0001‐AppendixS1.docx. [file ECE3-15-e71934-s001.docx]

# Appendix

**Table S1 The biological parameters of each functional group obtained from literatures**

| Function Group name | B_j_ (t/km^2^)^a^ | | | | | (P/B)_j_^b^  (year^-1^) | (Q/B)_j_^b^  (year^-1^) | e_j_^b^  ((P/Q)_j_) |
| --- | --- | --- | --- | --- | --- | --- | --- | --- |
|  | 1958 | 1980 | 1993 | 2009 | 2019 |  |  |  |
| Detritus | 145.30^d^ | 221.40^d^ | 471.70^d^ | 275.80^d^ | 378.70^d^ |  |  |  |
| Macrophytes | 225.40 | 397.99 | 369.75 | 114.45 | 212.02 | 2.25 |  |  |
| Phytoplankton | 0.60 | 0.90 | 1.94 | 4.19 | 0.85 | 185 |  |  |
| Large zooplankton | 0.34 | 1.02 | 2.50 | 0.85 | 1.05 | 25 | 270 | 0.09 |
| Meiofauna | 0.16 | 0.25 | 1.54 | 0.46 | 0.05 | 4.862^c^ | 97.24^c^ | 0.05^c^ |
| Mollusk | 11.78 | 5.87 | 4.50 | 2.81 | 33.35 | 3 | 9.75 | 0.31 |
| Herbivorous fish | 2.02 | 0.80 | 0.73 | 1.45 | 0.97 | 1.65 | 13.267 | 0.12 |
| Filter-feeding fish | 0.01 | 0.08 | 0.84 | 3.08 | 1.91 | 1.07^c^ | 6.114^c^ | 0.175^c^ |
| Fingerlings | 1.00 | 0.83 | 1.46 | 1.13 | 1.16 | 2.23 | 20.41 | 0.109 |
| Small omnivorous fish | 2.11 | 1.10 | 2.46 | 2.20 | 5.19 | 1.42 | 8.05 | 0.18 |
| Omnivorous fish | 1.06 | 0.55 | 1.23 | 1.10 | 1.80 | 1.99 | 11.874 | 0.17 |
| Carnivorous fish | 0.63 | 0.50 | 0.40 | 0.29 | 2.17 | 1.21 | 3.8 | 0.32 |

Notes: B_j_= Biomass of species j; (P/B)_j_= Production to Biomass ratio of species *j*, (Q/B)_j_ = Consumption to Biomass ratio of species *j*; e_j_= (P/Q)_j_ = Production to Consumption ratio, represent the efficiency of transformation of prey’s biomass into predator’s biomass.

1. The data come from Yang et al., (2022).
2. The data come from Zeng et al., (2021).
3. The data come from Guo et al., (2020).
4. The data come from Zhang et al., (2022).

**Table S2 Diet matrix of BYD Lake species from the 1950s to 2020s (obtained from Yang** **et al. (2022)**)

1. **1958 (unit: %).**

| **Groups** | Detr | SubM | Phyt | Zoop | Meio | Moll | HerF | FilF | Fing | SomF | LomF | CarF |
| --- | --- | --- | --- | --- | --- | --- | --- | --- | --- | --- | --- | --- |
| Detr | 0 | 0 | 0 | 74.05 | 79.2 | 67.4 | 1.2 | 1.2 | 13.8 | 20.3 | 15.4 | 0 |
| SubM | 0 | 0 | 0 | 0 | 1.2 | 10.7 | 96.3 | 0 | 13.9 | 57.4 | 20.7 | 0 |
| Phyt | 0 | 0 | 0 | 25.95 | 19.6 | 19.8 | 1.2 | 64.2 | 13.9 | 1.2 | 1.2 | 0 |
| Zoop | 0 | 0 | 0 | 0 | 0 | 2.1 | 1.3 | 34.6 | 37.9 | 19.9 | 1.2 | 0 |
| Meio | 0 | 0 | 0 | 0 | 0 | 0 | 0 | 0 | 1.2 | 1.2 | 1.2 | 0 |
| Moll | 0 | 0 | 0 | 0 | 0 | 0 | 0 | 0 | 19.3 | 0 | 57.8 | 3.96 |
| HerF | 0 | 0 | 0 | 0 | 0 | 0 | 0 | 0 | 0 | 0 | 0 | 3.95 |
| FilF | 0 | 0 | 0 | 0 | 0 | 0 | 0 | 0 | 0 | 0 | 0 | 2.19 |
| Fing | 0 | 0 | 0 | 0 | 0 | 0 | 0 | 0 | 0 | 0 | 2.5 | 38.5 |
| SomF | 0 | 0 | 0 | 0 | 0 | 0 | 0 | 0 | 0 | 0 | 0 | 32.1 |
| LomF | 0 | 0 | 0 | 0 | 0 | 0 | 0 | 0 | 0 | 0 | 0 | 19.3 |
| CarF | 0 | 0 | 0 | 0 | 0 | 0 | 0 | 0 | 0 | 0 | 0 | 0 |

1. **1980 (unit: %).**

| **Groups** | Detr | SubM | Phyt | Zoop | Meio | Moll | HerF | FilF | Fing | SomF | LomF | CarF |
| --- | --- | --- | --- | --- | --- | --- | --- | --- | --- | --- | --- | --- |
| Detr | 0 | 0 | 0 | 76.2 | 79.1 | 65.5 | 0.5 | 2.4 | 11.8 | 19.7 | 11.9 | 0 |
| SubM | 0 | 0 | 0 | 0 | 1.2 | 9.9 | 98.3 | 0 | 19.7 | 55.7 | 22.2 | 0 |
| Phyt | 0 | 0 | 0 | 23.8 | 19.7 | 22.2 | 0.6 | 55.1 | 14.8 | 2.4 | 2.4 | 0 |
| Zoop | 0 | 0 | 0 | 0 | 0 | 2.4 | 0.6 | 42.5 | 39.5 | 18.6 | 7.1 | 3.6 |
| Meio | 0 | 0 | 0 | 0 | 0 | 0 | 0 | 0 | 2.4 | 3.6 | 3.6 | 0 |
| Moll | 0 | 0 | 0 | 0 | 0 | 0 | 0 | 0 | 11.8 | 0 | 49.2 | 17.4 |
| HerF | 0 | 0 | 0 | 0 | 0 | 0 | 0 | 0 | 0 | 0 | 0 | 2.4 |
| FilF | 0 | 0 | 0 | 0 | 0 | 0 | 0 | 0 | 0 | 0 | 0 | 1.2 |
| Fing | 0 | 0 | 0 | 0 | 0 | 0 | 0 | 0 | 0 | 0 | 3.6 | 34.4 |
| SomF | 0 | 0 | 0 | 0 | 0 | 0 | 0 | 0 | 0 | 0 | 0 | 22.2 |
| LomF | 0 | 0 | 0 | 0 | 0 | 0 | 0 | 0 | 0 | 0 | 0 | 18.8 |
| CarF | 0 | 0 | 0 | 0 | 0 | 0 | 0 | 0 | 0 | 0 | 0 | 0 |

1. **1993 (unit:%).**

| **Groups** | Detr | SubM | Phyt | Zoop | Meio | Moll | HerF | FilF | Fing | SomF | LomF | CarF |
| --- | --- | --- | --- | --- | --- | --- | --- | --- | --- | --- | --- | --- |
| Detr | 0 | 0 | 0 | 76.1 | 80.2 | 64.4 | 0.3 | 1.2 | 12.1 | 22.1 | 11.2 | 0 |
| SubM | 0 | 0 | 0 | 0 | 1.2 | 12.2 | 99 | 0 | 18.4 | 56.1 | 21 | 0 |
| Phyt | 0 | 0 | 0 | 23.9 | 18.6 | 21 | 0.7 | 54.4 | 14.8 | 1.2 | 1.2 | 0 |
| Zoop | 0 | 0 | 0 | 0 | 0 | 2.4 | 0 | 44.4 | 40.1 | 17 | 19.8 | 18 |
| Meio | 0 | 0 | 0 | 0 | 0 | 0 | 0 | 0 | 2.4 | 3.6 | 3.6 | 0 |
| Moll | 0 | 0 | 0 | 0 | 0 | 0 | 0 | 0 | 12.2 | 0 | 39.6 | 7.2 |
| HerF | 0 | 0 | 0 | 0 | 0 | 0 | 0 | 0 | 0 | 0 | 0 | 2.4 |
| FilF | 0 | 0 | 0 | 0 | 0 | 0 | 0 | 0 | 0 | 0 | 0 | 3.6 |
| Fing | 0 | 0 | 0 | 0 | 0 | 0 | 0 | 0 | 0 | 0 | 3.6 | 36.6 |
| SomF | 0 | 0 | 0 | 0 | 0 | 0 | 0 | 0 | 0 | 0 | 0 | 21 |
| LomF | 0 | 0 | 0 | 0 | 0 | 0 | 0 | 0 | 0 | 0 | 0 | 11.2 |
| CarF | 0 | 0 | 0 | 0 | 0 | 0 | 0 | 0 | 0 | 0 | 0 | 0 |

1. **2009 (unit: %).**

| **Groups** | Detr | SubM | Phyt | Zoop | Meio | Moll | HerF | FilF | Fing | SomF | LomF | CarF |
| --- | --- | --- | --- | --- | --- | --- | --- | --- | --- | --- | --- | --- |
| Detr | 0 | 0 | 0 | 75.7 | 79.4 | 63.2 | 1.5 | 2.4 | 13.7 | 29 | 10.1 | 0 |
| SubM | 0 | 0 | 0 | 0 | 1.2 | 3.6 | 97.1 | 0 | 19.5 | 38.8 | 19.4 | 0 |
| Phyt | 0 | 0 | 0 | 24.3 | 19.4 | 31.3 | 1.4 | 62.5 | 30.7 | 19.4 | 19.4 | 4.8 |
| Zoop | 0 | 0 | 0 | 0 | 0 | 1.9 | 0 | 35.1 | 22.5 | 9.2 | 13 | 0 |
| Meio | 0 | 0 | 0 | 0 | 0 | 0 | 0 | 0 | 6.8 | 3.6 | 4.8 | 0 |
| Moll | 0 | 0 | 0 | 0 | 0 | 0 | 0 | 0 | 6.8 | 0 | 32.1 | 4.8 |
| HerF | 0 | 0 | 0 | 0 | 0 | 0 | 0 | 0 | 0 | 0 | 0 | 4.8 |
| FilF | 0 | 0 | 0 | 0 | 0 | 0 | 0 | 0 | 0 | 0 | 0 | 4.8 |
| Fing | 0 | 0 | 0 | 0 | 0 | 0 | 0 | 0 | 0 | 0 | 1.2 | 34.9 |
| SomF | 0 | 0 | 0 | 0 | 0 | 0 | 0 | 0 | 0 | 0 | 0 | 34.9 |
| LomF | 0 | 0 | 0 | 0 | 0 | 0 | 0 | 0 | 0 | 0 | 0 | 11 |
| CarF | 0 | 0 | 0 | 0 | 0 | 0 | 0 | 0 | 0 | 0 | 0 | 0 |

1. **2019 (unit: %).**

| **Groups** | Detr | SubM | Phyt | Zoop | Meio | Moll | HerF | FilF | Fing | SomF | LomF | CarF |
| --- | --- | --- | --- | --- | --- | --- | --- | --- | --- | --- | --- | --- |
| Detr | 0 | 0 | 0 | 11.76 | 60.37 | 58.51 | 11.04 | 25.52 | 6.73 | 5.18 | 13.04 | 0 |
| SubM | 0 | 0 | 0 | 0 | 18.65 | 9.39 | 33.88 | 0 | 27.93 | 28.51 | 18.31 | 0 |
| Phyt | 0 | 0 | 0 | 88.24 | 20.98 | 19.11 | 32.31 | 28.08 | 11.14 | 9.96 | 18.22 | 0 |
| Zoop | 0 | 0 | 0 | 0 | 0 | 12.99 | 22.76 | 46.41 | 19.1 | 26.86 | 15.6 | 11.12 |
| Meio | 0 | 0 | 0 | 0 | 0 | 0 | 0 | 0 | 19.37 | 29.48 | 12.02 | 0 |
| Moll | 0 | 0 | 0 | 0 | 0 | 0 | 0 | 0 | 15.74 | 0 | 12.17 | 13.6 |
| HerF | 0 | 0 | 0 | 0 | 0 | 0 | 0 | 0 | 0 | 0 | 0 | 7.69 |
| FilF | 0 | 0 | 0 | 0 | 0 | 0 | 0 | 0 | 0 | 0 | 0 | 18.6 |
| Fing | 0 | 0 | 0 | 0 | 0 | 0 | 0 | 0 | 0 | 0 | 10.63 | 21.44 |
| SomF | 0 | 0 | 0 | 0 | 0 | 0 | 0 | 0 | 0 | 0 | 0 | 20.56 |
| LomF | 0 | 0 | 0 | 0 | 0 | 0 | 0 | 0 | 0 | 0 | 0 | 6.99 |
| CarF | 0 | 0 | 0 | 0 | 0 | 0 | 0 | 0 | 0 | 0 | 0 | 0 |

Notes: (1)CarF-Carnivorous fish; LomF-Large omnivorous fish; SomF-Small omnivorous fish; Fing-Fingerlings; FilF-Filter-feeding fish; HerF-Herbivorous fish; MolL-Mollusks; Meio-Meiofauna; ZooP-Zooplankton; PhyT-Phytoplankton; SubM-Submerged macrophytes; Detr-Detritus.

(2) The groups as consumers run across the columns and groups as prey run down the rows

**Table S3 The estimated outputs from the Ecopath model of BYD Lake from the 1950s to 2020s**

| No. | Group name | Trophic level | Biomass  (g/m^2) | P / B  (/year) | Q /B  (/year) | EE | P / Q  (/year) | Non-predation  mortality(/year) |
| --- | --- | --- | --- | --- | --- | --- | --- | --- |
| 1 | CarF | 3.37 | 0.50 | 1.21 | 3.80 | 0.00 | 0.32 | 1.21 |
| 2 | LomF | 2.67 | 0.55 | 1.99 | 11.87 | 0.33 | 0.17 | 1.56 |
| 3 | SomF | 2.22 | 1.10 | 1.42 | 8.05 | 0.27 | 0.18 | 1.06 |
| 4 | Fing | 2.54 | 0.83 | 2.23 | 20.41 | 0.48 | 0.11 | 0.94 |
| 5 | FilF | 2.43 | 0.80 | 1.07 | 6.11 | 0.03 | 0.18 | 0.98 |
| 6 | HerF | 2.01 | 0.80 | 1.65 | 13.27 | 0.03 | 0.12 | 1.60 |
| 7 | MolL | 2.02 | 5.87 | 3.00 | 9.75 | 0.31 | 0.31 | 2.05 |
| 8 | Meio | 2.00 | 0.25 | 4.86 | 97.24 | 0.79 | 0.05 | 1.63 |
| 9 | ZooP | 2.00 | 1.02 | 25.00 | 270.00 | 0.49 | 0.09 | 1.47 |
| 10 | PhyT | 1.00 | 0.90 | 185.00 |  | 0.53 |  | 57.88 |
| 11 | SubM | 1.00 | 397.99 | 2.25 |  | 0.03 |  | 2.01 |
| 12 | Detr | 1.00 | 221.40 |  |  | 0.26 |  | 1.00 |

(a) 1958.

(b) 1980.

| No. | Group name | Trophic level | Biomass  (g/m^2) | P / B  (/year) | Q /B  (/year) | EE | P / Q  (/year) | Non-predation  mortality(/year) |
| --- | --- | --- | --- | --- | --- | --- | --- | --- |
| 1 | CarF | 3.43 | 0.63 | 1.21 | 3.80 | 0.00 | 0.32 | 1.21 |
| 2 | LomF | 2.66 | 1.06 | 1.99 | 11.87 | 0.22 | 0.17 | 1.34 |
| 3 | SomF | 2.21 | 2.11 | 1.42 | 8.05 | 0.26 | 0.18 | 1.04 |
| 4 | Fing | 2.58 | 1.00 | 2.23 | 20.41 | 0.58 | 0.11 | 1.16 |
| 5 | FilF | 2.35 | 0.13 | 1.07 | 6.11 | 0.34 | 0.18 | 1.04 |
| 6 | HerF | 2.01 | 2.02 | 1.65 | 13.27 | 0.03 | 0.12 | 1.59 |
| 7 | MolL | 2.02 | 11.78 | 3.00 | 9.75 | 0.32 | 0.31 | 2.06 |
| 8 | Meio | 2.00 | 0.16 | 4.86 | 97.24 | 0.66 | 0.05 | 1.02 |
| 9 | ZooP | 2.00 | 0.64 | 25.00 | 270.00 | 0.89 | 0.09 | 12.86 |
| 10 | PhyT | 1.00 | 0.60 | 185.00 |  | 0.67 |  | 86.47 |
| 11 | SubM | 1.00 | 225.40 | 2.25 |  | 0.11 |  | 2.18 |
| 12 | Detr | 1.00 | 145.30 |  |  | 0.37 |  | 1.00 |

(c) 1993.

| No. | Group name | Trophic level | Biomass  (g/m^2) | P / B  (/year) | Q /B  (/year) | EE | P / Q  (/year) | Non-predation  mortality(/year) |
| --- | --- | --- | --- | --- | --- | --- | --- | --- |
| 1 | CarF | 3.34 | 0.40 | 1.21 | 3.80 | 0.00 | 0.32 | 1.21 |
| 2 | LomF | 2.70 | 1.23 | 1.99 | 11.87 | 0.07 | 0.17 | 1.85 |
| 3 | SomF | 2.21 | 2.46 | 1.42 | 8.05 | 0.09 | 0.18 | 1.29 |
| 4 | Fing | 2.55 | 1.46 | 2.23 | 20.41 | 0.35 | 0.11 | 1.45 |
| 5 | FilF | 2.44 | 0.84 | 1.07 | 6.11 | 0.07 | 0.18 | 1.00 |
| 6 | HerF | 2.00 | 0.73 | 1.65 | 13.27 | 0.03 | 0.12 | 1.61 |
| 7 | MolL | 2.02 | 4.50 | 3.00 | 9.75 | 0.70 | 0.31 | 0.89 |
| 8 | Meio | 2.00 | 1.54 | 4.86 | 97.24 | 0.26 | 0.05 | 3.58 |
| 9 | ZooP | 2.00 | 2.50 | 25.00 | 270.00 | 0.35 | 0.09 | 16.30 |
| 10 | PhyT | 1.00 | 1.94 | 185.00 |  | 0.58 |  | 78.03 |
| 11 | SubM | 1.00 | 369.75 | 2.25 |  | 0.04 |  | 2.15 |
| 12 | Detr | 1.00 | 471.70 |  |  | 0.56 |  | 1.00 |

(d) 2009.

| No. | Group name | Trophic level | Biomass  (g/m^2) | P / B  (/year) | Q /B  (/year) | EE | P / Q  (/year) | Non-predation  mortality(/year) |
| --- | --- | --- | --- | --- | --- | --- | --- | --- |
| 1 | CarF | 3.20 | 0.29 | 1.21 | 3.80 | 0.00 | 0.32 | 1.21 |
| 2 | LomF | 2.52 | 1.10 | 1.99 | 11.87 | 0.06 | 0.17 | 1.88 |
| 3 | SomF | 2.13 | 2.20 | 1.42 | 8.05 | 0.12 | 0.18 | 1.25 |
| 4 | Fing | 2.36 | 1.13 | 2.23 | 20.41 | 0.21 | 0.11 | 1.78 |
| 5 | FilF | 2.35 | 2.53 | 1.07 | 6.11 | 0.02 | 0.18 | 1.05 |
| 6 | HerF | 2.00 | 1.45 | 1.65 | 13.27 | 0.02 | 0.12 | 1.61 |
| 7 | MolL | 2.02 | 2.81 | 3.00 | 9.75 | 0.69 | 0.31 | 0.91 |
| 8 | Meio | 2.00 | 1.46 | 4.86 | 97.24 | 0.40 | 0.05 | 1.55 |
| 9 | ZooP | 2.00 | 0.85 | 25.00 | 270.00 | 0.68 | 0.09 | 7.97 |
| 10 | PhyT | 1.00 | 4.19 | 185.00 |  | 0.15 |  | 62.32 |
| 11 | SubM | 1.00 | 114.45 | 2.25 |  | 0.14 |  | 1.95 |
| 12 | Detr | 1.00 | 275.80 |  |  | 0.31 |  | 1.00 |

1. 2019.

| No. | Group name | Trophic level | Biomass  (g/m^2) | P / B  (/year) | Q /B  (/year) | EE | P / Q  (/year) | Non-predation  mortality(/year) |
| --- | --- | --- | --- | --- | --- | --- | --- | --- |
| 1 | CarF | 3.40 | 2.17 | 1.21 | 3.80 | 0.00 | 0.32 | 1.21 |
| 2 | LomF | 2.59 | 1.80 | 1.99 | 11.87 | 0.16 | 0.17 | 1.67 |
| 3 | SomF | 2.56 | 5.19 | 1.42 | 8.05 | 0.23 | 0.18 | 1.09 |
| 4 | Fing | 2.56 | 2.16 | 2.23 | 20.41 | 0.84 | 0.11 | 0.35 |
| 5 | FilF | 2.46 | 1.91 | 1.07 | 6.11 | 0.76 | 0.18 | 0.26 |
| 6 | HerF | 2.23 | 0.97 | 1.65 | 13.27 | 0.41 | 0.12 | 0.98 |
| 7 | MolL | 2.13 | 41.35 | 3.00 | 9.75 | 0.09 | 0.31 | 2.74 |
| 8 | Meio | 2.00 | 1.23 | 20.86 | 97.24 | 0.90 | 0.21 | 2.12 |
| 9 | ZooP | 2.00 | 3.85 | 25.00 | 270.00 | 0.88 | 0.09 | 3.00 |
| 10 | PhyT | 1.00 | 5.85 | 185.00 |  | 0.96 |  | 7.78 |
| 11 | SubM | 1.00 | 212.02 | 2.25 |  | 0.19 |  | 1.82 |
| 12 | Detr | 1.00 | 378.70 |  |  | 0.49 |  | 1.00 |

Notes: CarF-Carnivorous fish; LomF-Large omnivorous fish; SomF-Small omnivorous fish; Fing-Fingerlings; FilF-Filter-feeding fish; HerF-Herbivorous fish; MolL-Mollusks; Meio-Meiofauna; ZooP-Zooplankton; PhyT-Phytoplankton; SubM-Submerged macrophytes; Detr-Detritus.

**Table S4 The Jacobian (Interaction strength) matrix of BYD Lake with and without considering detritus flux**

(a)1958.

| Item | Detr | SubM | Phyt | Zoop | Meio | Moll | HerF | FilF | Fing | SomF | LomF | CarF |
| --- | --- | --- | --- | --- | --- | --- | --- | --- | --- | --- | --- | --- |
| Ddetr | 0.0000 | 0.9640 | 0.6459 | -277.1344 | -94.5706 | -7.0330 | 0.8707 | -3.8717 | -7.0786 | -0.6402 | -2.6217 | 1.0000 |
| Gdetr | 0.0000 | 0.0000 | 0.0000 | -277.3764 | -94.9122 | -7.7567 | -0.1096 | -4.5374 | -7.5992 | -1.4634 | -3.4517 | 0.000 |
| SubM | 0.0000 | 0.0000 | 0.0000 | 0.0000 | -2.2308 | -1.9102 | -13.6395 | 0.0000 | -11.8739 | -6.4188 | -7.1974 | 0.0000 |
| Phyt | 0.0000 | 0.0000 | 0.0000 | -0.4014 | -0.0970 | -0.0094 | -0.0005 | -1.0024 | -0.0316 | -0.0004 | -0.0011 | 0.0000 |
| Zoop | 0.1096 | 0.0000 | 0.0384 | 0.0000 | 0.0000 | -0.0011 | -0.0005 | -0.5745 | -0.0916 | -0.0063 | -0.0012 | 0.0000 |
| Meio | 0.0051 | 0.0001 | 0.0013 | 0.0000 | 0.0000 | 0.0000 | 0.0000 | 0.0000 | -0.0007 | -0.0001 | -0.0003 | 0.0000 |
| Moll | 0.1949 | 0.0309 | 0.0573 | 0.0061 | 0.0000 | 0.0000 | 0.0000 | 0.0000 | -0.8616 | 0.0000 | -1.0503 | -0.9672 |
| HerF | 0.0002 | 0.0147 | 0.0002 | 0.0002 | 0.0000 | 0.0000 | 0.0000 | 0.0000 | 0.0000 | 0.0000 | 0.0000 | -0.1653 |
| FilF | 0.0029 | 0.0000 | 0.1550 | 0.0835 | 0.0000 | 0.0000 | 0.0000 | 0.0000 | 0.0000 | 0.0000 | 0.0000 | -0.0241 |
| Fing | 0.0057 | 0.0057 | 0.0057 | 0.0157 | 0.0005 | 0.0080 | 0.0000 | 0.0000 | 0.0000 | 0.0000 | -0.0039 | -0.7982 |
| SomF | 0.0038 | 0.0108 | 0.0002 | 0.0037 | 0.0002 | 0.0000 | 0.0000 | 0.0000 | 0.0000 | 0.0000 | 0.0000 | -1.4043 |
| LomF | 0.0043 | 0.0057 | 0.0003 | 0.0003 | 0.0003 | 0.0160 | 0.0000 | 0.0000 | 0.0007 | 0.0000 | 0.0000 | -0.4222 |
| CarF | 0.0000 | 0.0000 | 0.0000 | 0.0000 | 0.0000 | 0.0164 | 0.0164 | 0.0091 | 0.1596 | 0.1331 | 0.0800 | 0.0000 |

(b)1980.

| Item | Detr | SubM | Phyt | Zoop | Meio | Moll | HerF | FilF | Fing | SomF | LomF | CarF |
| --- | --- | --- | --- | --- | --- | --- | --- | --- | --- | --- | --- | --- |
| DDetr | 0.0000 | 0.9143 | 0.5202 | -277.2240 | -94.2237 | -6.8731 | 0.9365 | -4.5400 | -4.5575 | -0.5070 | -1.7985 | 1.0000 |
| GDetr | 0.0000 | 0.0000 | 0.0000 | -277.4244 | -94.5651 | -7.6006 | -0.0388 | -5.2027 | -5.0536 | -1.2959 | -2.6198 | 0.0000 |
| SubM | 0.0000 | 0.0000 | 0.0000 | 0.0000 | -2.5789 | -2.0651 | -13.7108 | 0.0000 | -15.1662 | -6.5865 | -8.7857 | 0.0000 |
| Phyt | 0.0000 | 0.0000 | 0.0000 | -0.3534 | -0.0961 | -0.0105 | -0.0002 | -0.4872 | -0.0259 | -0.0006 | -0.0022 | 0.0000 |
| Zoop | 0.1150 | 0.0000 | 0.0359 | 0.0000 | 0.0000 | -0.0013 | -0.0002 | -0.4244 | -0.0779 | -0.0056 | -0.0072 | -0.0808 |
| Meio | 0.0053 | 0.0001 | 0.0013 | 0.0000 | 0.0000 | 0.0000 | 0.0000 | 0.0000 | -0.0012 | -0.0003 | -0.0009 | 0.0000 |
| Moll | 0.0625 | 0.0094 | 0.0212 | 0.0023 | 0.0000 | 0.0000 | 0.0000 | 0.0000 | -0.1340 | 0.0000 | -0.2872 | -2.2476 |
| HerF | 0.0000 | 0.0033 | 0.00002 | 0.0000 | 0.0000 | 0.0000 | 0.0000 | 0.0000 | 0.0000 | 0.0000 | 0.0000 | -0.0423 |
| FilF | 0.0033 | 0.0000 | 0.0755 | 0.0583 | 0.0000 | 0.0000 | 0.0000 | 0.0000 | 0.0000 | 0.0000 | 0.0000 | -0.0211 |
| Fing | 0.0021 | 0.0034 | 0.0026 | 0.0069 | 0.0004 | 0.0021 | 0.0000 | 0.0000 | 0.0000 | 0.0000 | -0.0030 | -0.6245 |
| SomF | 0.0012 | 0.0033 | 0.0001 | 0.0011 | 0.0002 | 0.0000 | 0.0000 | 0.0000 | 0.0000 | 0.0000 | 0.0000 | -0.5374 |
| LomF | 0.0011 | 0.0021 | 0.0002 | 0.0007 | 0.0003 | 0.0046 | 0.0000 | 0.0000 | 0.0003 | 0.0000 | 0.0000 | -0.2275 |
| CarF | 0.0000 | 0.0000 | 0.0000 | 0.0127 | 0.0000 | 0.0613 | 0.0085 | 0.0042 | 0.1211 | 0.0782 | 0.0662 | 0.0000 |

| Item | Detr | SubM | Phyt | Zoop | Meio | Moll | HerF | FilF | Fing | SomF | LomF | CarF |
| --- | --- | --- | --- | --- | --- | --- | --- | --- | --- | --- | --- | --- |
| DDetr | 0.0000 | 0.9195 | 0.5208 | -277.1990 | -95.6801 | -7.7098 | 0.9207 | -3.7594 | -8.7198 | -1.8627 | -3.8570 | 1 |
| GDetr | 0.0000 | 0.0000 | 0.0000 | -277.4204 | -96.0224 | -8.4149 | -0.0529 | -4.4248 | -9.1957 | -2.6343 | -4.6538 | 0.0000 |
| SubM | 0.0000 | 0.0000 | 0.0000 | 0.0000 | -1.1262 | -1.2496 | -13.6965 | 0.0000 | -10.9611 | -5.2418 | -6.8399 | 0.0000 |
| Phyt | 0.0000 | 0.0000 | 0.0000 | -0.3574 | -0.0914 | -0.0113 | -0.0005 | -0.8229 | -0.0461 | -0.0006 | -0.0020 | 0.0000 |
| Zoop | 0.1322 | 0.0000 | 0.0415 | 0.0000 | 0.0000 | -0.0017 | 0.0000 | -0.8667 | -0.1613 | -0.0107 | -0.0436 | -0.8456 |
| Meio | 0.0156 | 0.0002 | 0.0036 | 0.0000 | 0.0000 | 0.0000 | 0.0000 | 0.0000 | -0.0059 | -0.0014 | -0.0049 | 0.0000 |
| Moll | 0.0249 | 0.0047 | 0.0081 | 0.0009 | 0.0000 | 0.0000 | 0.0000 | 0.0000 | -0.0885 | 0.0000 | -0.1570 | -0.6099 |
| HerF | 0.0000 | 0.0032 | 0.0000 | 0.0000 | 0.0000 | 0.0000 | 0.0000 | 0.0000 | 0.0000 | 0.0000 | 0.0000 | -0.0327 |
| FilF | 0.0014 | 0.0000 | 0.0625 | 0.0510 | 0.0000 | 0.0000 | 0.0000 | 0.0000 | 0.0000 | 0.0000 | 0.0000 | -0.0569 |
| Fing | 0.0031 | 0.0047 | 0.0038 | 0.0103 | 0.0006 | 0.0031 | 0.0000 | 0.0000 | 0.0000 | 0.0000 | -0.0046 | -1.0040 |
| SomF | 0.0025 | 0.0063 | 0.0001 | 0.0019 | 0.0004 | 0.0000 | 0.0000 | 0.0000 | 0.0000 | 0.0000 | 0.0000 | -0.9727 |
| LomF | 0.0021 | 0.0039 | 0.0002 | 0.0037 | 0.0007 | 0.0073 | 0.0000 | 0.0000 | 0.0007 | 0.0000 | 0.0000 | -0.2594 |
| CarF | 0.0000 | 0.0000 | 0.0000 | 0.0433 | 0.0000 | 0.0173 | 0.0058 | 0.0087 | 0.0881 | 0.0506 | 0.0270 | 0.0000 |

(c)1993.

(d)2009.

| Item | Detr | SubM | Phyt | Zoop | Meio | Moll | HerF | FilF | Fing | SomF | LomF | CarF |
| --- | --- | --- | --- | --- | --- | --- | --- | --- | --- | --- | --- | --- |
| DDetr | 0.0000 | 0.9600 | 0.6491 | -276.1810 | -95.9308 | -8.6773 | 0.4826 | -3.5793 | -12.045 | -4.2531 | -5.4870 | 1.0000 |
| GDetr | 0.0000 | 0.0000 | 0.0000 | -276.4313 | -96.2792 | -9.3842 | -0.4932 | -4.2452 | -12.5084 | -5.0359 | -6.2805 | 0.0000 |
| SubM | 0.0000 | 0.0000 | 0.0000 | 0.0000 | -0.6038 | -0.2218 | -13.2498 | 0.0000 | -7.3881 | -2.7959 | -5.0060 | 0.0000 |
| Phyt | 0.0000 | 0.0000 | 0.0000 | -1.3465 | -0.3570 | -0.0705 | -0.0070 | -1.6775 | -0.4253 | -0.0511 | -0.1831 | -0.4203 |
| Zoop | 0.0768 | 0.0000 | 0.0246 | 0.0000 | 0.0000 | -0.0009 | 0.0000 | -0.1916 | -0.0634 | -0.0049 | -0.0249 | 0.0000 |
| Meio | 0.0080 | 0.0001 | 0.0019 | 0.0000 | 0.0000 | 0.0000 | 0.0000 | 0.0000 | -0.0103 | -0.0010 | -0.0049 | 0.0000 |
| Moll | 0.0296 | 0.0017 | 0.0147 | 0.0009 | 0.0000 | 0.0000 | 0.0000 | 0.0000 | -0.0633 | 0.0000 | -0.2034 | -0.2822 |
| HerF | 0.0008 | 0.0201 | 0.0003 | 0.0000 | 0.0000 | 0.0000 | 0.0000 | 0.0000 | 0.0000 | 0.0000 | 0.0000 | -0.1454 |
| FilF | 0.0068 | 0.0000 | 0.1775 | 0.0997 | 0.0000 | 0.0000 | 0.0000 | 0.0000 | 0.0000 | 0.0000 | 0.0000 | -0.2541 |
| Fing | 0.0056 | 0.0079 | 0.0125 | 0.0092 | 0.0028 | 0.0028 | 0.0000 | 0.0000 | 0.0000 | 0.0000 | -0.0031 | -0.8237 |
| SomF | 0.0072 | 0.0097 | 0.0048 | 0.0023 | 0.0009 | 0.0000 | 0.0000 | 0.0000 | 0.0000 | 0.0000 | 0.0000 | -1.6028 |
| LomF | 0.0043 | 0.0082 | 0.0082 | 0.0055 | 0.0020 | 0.0135 | 0.0000 | 0.0000 | 0.0005 | 0.0000 | 0.0000 | -0.2527 |
| CarF | 0.0000 | 0.0000 | 0.0094 | 0.0000 | 0.0000 | 0.0094 | 0.0094 | 0.0094 | 0.0685 | 0.0685 | 0.0216 | 0.0000 |

(e)2019.

| Item | Detr | SubM | Phyt | Zoop | MicZ | Moll | HerF | FilF | Fing | SomF | LomF | CarF |
| --- | --- | --- | --- | --- | --- | --- | --- | --- | --- | --- | --- | --- |
| DDetr | 0.0000 | 0.9814 | 1.5667 | -272.9180 | -82.4620 | -8.1459 | -4.0496 | -5.3686 | -5.2266 | -1.1407 | -5.3759 | 1.0000 |
| GDetr | 0.0000 | 0.0000 | 0.0000 | -273.1559 | -82.8475 | -8.8688 | -5.0362 | -6.0687 | -5.6976 | -1.9251 | -6.1710 | 0.0000 |
| SubM | 0.0000 | 0.0000 | 0.0000 | 0.0000 | -14.3276 | -0.7966 | -8.6519 | 0.0000 | -13.2385 | -5.9265 | -4.8499 | 0.0000 |
| Phyt | 0.0000 | 0.0000 | 0.0000 | -4.6218 | -0.0649 | -0.0065 | -0.0332 | -0.0151 | -0.0213 | -0.0083 | -0.0194 | 0.0000 |
| Zoop | 0.0680 | 0.0000 | 0.5105 | 0.0000 | 0.0000 | -0.0054 | -0.0287 | -0.0305 | -0.0447 | -0.0276 | -0.0204 | -0.0579 |
| MicZ | 0.0005 | 0.0002 | 0.0002 | 0.0000 | 0.0000 | 0.0000 | 0.0000 | 0.0000 | -0.0021 | -0.0014 | -0.0007 | 0.0000 |
| Moll | 0.3002 | 0.0482 | 0.0980 | 0.0666 | 0.0000 | 0.0000 | 0.0000 | 0.0000 | -1.4545 | 0.0000 | -0.6290 | -2.7940 |
| HerF | 0.0015 | 0.0047 | 0.0045 | 0.0032 | 0.0000 | 0.0000 | 0.0000 | 0.0000 | 0.0000 | 0.0000 | 0.0000 | -0.0370 |
| FilF | 0.0054 | 0.0000 | 0.0059 | 0.0097 | 0.0000 | 0.0000 | 0.0000 | 0.0000 | 0.0000 | 0.0000 | 0.0000 | -0.1763 |
| Fing | 0.0019 | 0.0079 | 0.0031 | 0.0054 | 0.0055 | 0.0044 | 0.0000 | 0.0000 | 0.0000 | 0.0000 | -0.0154 | -0.1233 |
| SomF | 0.0047 | 0.0261 | 0.0091 | 0.0246 | 0.0270 | 0.0000 | 0.0000 | 0.0000 | 0.0000 | 0.0000 | 0.0000 | -0.5303 |
| LomF | 0.0050 | 0.0070 | 0.0070 | 0.0060 | 0.0046 | 0.0047 | 0.0000 | 0.0000 | 0.0041 | 0.0000 | 0.0000 | -0.0625 |
| CarF | 0.0000 | 0.0000 | 0.0000 | 0.0384 | 0.0000 | 0.0469 | 0.0265 | 0.0642 | 0.0739 | 0.0709 | 0.0241 | 0.0000 |

Notes:(1)CarF-Carnivorous fish; LomF-Large omnivorous fish; SomF-Small omnivorous fish; Fing-Fingerlings; FilF-Filter-feeding fish; HerF-Herbivorous fish; MolL-Mollusks; Meio-Meiofauna; ZooP-Zooplankton; PhyT-Phytoplankton; SubM-Submerged macrophytes; DDetr-Detritus in detritus-based food web (with consideration of detritus flux); GDetr- Detritus in phytoplankton-based food web (without consideration of detritus flux)

(2)The affected groups (impacted groups) run down the rows and the impacting groups run across the columns.
